# Deciphering the Interactions of Bioactive Compounds in Selected Traditional Medicinal Plants against Alzheimer’s Diseases via Pharmacophore Modeling, Auto-QSAR, and Molecular Docking Approaches

**DOI:** 10.3390/molecules26071996

**Published:** 2021-04-01

**Authors:** Oluwafemi Adeleke Ojo, Adebola Busola Ojo, Charles Okolie, Mary-Ann Chinyere Nwakama, Matthew Iyobhebhe, Ikponmwosa Owen Evbuomwan, Charles Obiora Nwonuma, Rotdelmwa Filibus Maimako, Abayomi Emmanuel Adegboyega, Odunayo Anthonia Taiwo, Khalaf F. Alsharif, Gaber El-Saber Batiha

**Affiliations:** 1 Medicinal Biochemistry and Biochemical Toxicology Group, Department of Biochemistry, Landmark University, Omu-Aran PMB 1001, Nigeria; adamaryann62@gmail.com (M.-A.C.N.); iyobhebhematthew@gmail.com (M.I.); nwonuma.charles@lmu.edu.ng (C.O.N.); maimako.rotdelmwa@lmu.edu.ng (R.F.M.); 2 Department of Biochemistry, Faculty of Sciences, Ekiti State University, Ado-Ekiti PMB 5363, Nigeria; adebolaojo04@gmail.com; 3 Department of Microbiology, Landmark University, Omu-Aran PMB 1001, Nigeria; charles.okolie@lmu.edu.ng (C.O.); evbuomwan.ikponmwosa@lmu.edu.ng (I.O.E.); 4 Department of Biochemistry, Faculty of Basic Medical Science, University of Jos, Jos PMB 2084, Nigeria; abayomiadegboyega5@gmail.com; 5 Department of Biochemistry, Chrisland University, Abeokuta PMB 2131, Nigeria; odunayotaiwo25@gmail.com; 6 Department of Clinical Laboratory Sciences, College of Applied Medical Sciences, Taif University, P.O. Box 11099, Taif 21944, Saudi Arabia; alsharif@tu.edu.sa; 7 Department of Pharmacology and Therapeutics, Faculty of Veterinary Medicine, Damanhour University, Damanhour, AlBeheira 22511, Egypt; gaberbatiha@gmail.com

**Keywords:** Alzheimer’s, pharmacophore modeling, QSAR, molecular docking, bioactive compounds, neurodegenerative diseases

## Abstract

Neurodegenerative diseases, for example Alzheimer’s, are perceived as driven by hereditary, cellular, and multifaceted biochemical actions. Numerous plant products, for example flavonoids, are documented in studies for having the ability to pass the blood-brain barrier and moderate the development of such illnesses. Computer-aided drug design (CADD) has achieved importance in the drug discovery world; innovative developments in the aspects of structure identification and characterization, bio-computational science, and molecular biology have added to the preparation of new medications towards these ailments. In this study we evaluated nine flavonoid compounds identified from three medicinal plants, namely *T. diversifolia*, *B. sapida,* and *I. gabonensis* for their inhibitory role on acetylcholinesterase (AChE), butyrylcholinesterase (BChE) and monoamine oxidase (MAO) activity, using pharmacophore modeling, auto-QSAR prediction, and molecular studies, in comparison with standard drugs. The results indicated that the pharmacophore models produced from structures of AChE, BChE and MAO could identify the active compounds, with a recuperation rate of the actives found near 100% in the complete ranked decoy database. Moreso, the robustness of the virtual screening method was accessed by well-established methods including enrichment factor (EF), receiver operating characteristic curve (ROC), Boltzmann-enhanced discrimination of receiver operating characteristic (BEDROC), and area under accumulation curve (AUAC). Most notably, the compounds’ pIC_50_ values were predicted by a machine learning-based model generated by the AutoQSAR algorithm. The generated model was validated to affirm its predictive model. The best models achieved for AChE, BChE and MAO were models kpls_radial_17 (R^2^ = 0.86 and Q^2^ = 0.73), pls_38 (R^2^ = 0.77 and Q^2^ = 0.72), kpls_desc_44 (R^2^ = 0.81 and Q^2^ = 0.81) and these externally validated models were utilized to predict the bioactivities of the lead compounds. The binding affinity results of the ligands against the three selected targets revealed that luteolin displayed the highest affinity score of −9.60 kcal/mol, closely followed by apigenin and ellagic acid with docking scores of −9.60 and −9.53 kcal/mol, respectively. The least binding affinity was attained by gallic acid (−6.30 kcal/mol). The docking scores of our standards were −10.40 and −7.93 kcal/mol for donepezil and galanthamine, respectively. The toxicity prediction revealed that none of the flavonoids presented toxicity and they all had good absorption parameters for the analyzed targets. Hence, these compounds can be considered as likely leads for drug improvement against the same.

## 1. Introduction

*Tithonia diversifolia* (Hemsl.) A. Gray is recognized as a curative herb employed in treating many infections [1]. It is commonly known as Mexican sunflower (in English), bush helianthus or *sepeleba* (in Yoruba). Numerous reports exist of its anti-Alzheimer, anti-diabetic, anti-inflammatory, antimicrobial, antimalaria, and anticarcinogenic effects [1]. The leaves contain active anti-inflammatory constituents including diversifolin and tirotundin [2] and gallic acid, chlorogenic acid, caffeic acid, *p*-coumaric acid, and apigenin active against cognitive dysfunction [3]. Furthermore, inhibitory and antioxidant properties of *T. diversifolia* extract on certain pro-oxidant mediated lipid peroxidation effects in the brain of rats was documented [4]. 

*Blighia sapida* K.D. Koenig, usually referred to as ‘Akee apple’, belongs to *Sapindaceae* plant family. *B. sapida* is a therapeutic herb generally employed by traditional medical practitioners and extremely appreciated in Africa for the treatment of numerous disorders [5]. It was reported to contain alkaloids, saponins, cardiac glycosides, reducing sugars and starches [6,7]. The extracts of *B. sapida* have been documented to show anti-microbial activity towards *S. aureus* and *B. subtilis*, antidiabetic and anti-Alzheimer’s activities, respectively [5,8,9,10,11].

*Irvingia gabonensis* (Aubry-Lecomte ex O’Rorke) Baill. (*Irvingiaceae*) is an ancestral fruit tree predominant in Africa and recognized primarily as a domesticated plant [12]. In Nigeria, it is referred to as bush mango. In some parts of Africa, the plant occurs freely and is a commonly used tropical African tree. Local names include Ogbono (Igbo) and Goron biri (Hausa). The seed is used as a food substitute. The stem bark is used in herbal medicine albeit without any scientific justification in managing numerous neurodegenerative disorders after macerating in dry gin [13]. It has been revealed that *I. gabonensis* extract could play a key part in the reduction of the neurotoxicological damage triggered by heavy metals. It also has pharmacological activities that include hepatoprotective [14], nephroprotective properties and anti-Alzheimer’s activity [15,16].

Alzheimer’s disease (AD) is exemplified by cognitive decline and several other intellectual damage symptoms. It is presently the most challenging advanced neurodegenerative diseases to manage [17,18,19]. Over the years, numerous hypotheses on the pathogenesis of AD have been suggested, including the amyloid cascade, tau protein, oxidative and cholinergic hypothesis [20]. Amongst these hypotheses, the cholinergic one indicating that reduced levels of the neurotransmitter acetylcholine in explicit areas of the cerebrum trigger learning and memory dysfunctions has become the most generally accepted hypothesis. A possible way to treat AD is thus to restore the levels of acetylcholine via reversible inhibitors to hinder acetylcholinesterase (AChE) and butyrylcholinesterase (BuChE) [16,20,21]. Besides, monoamine oxidases (MAO) perform an important physiological function in neurotransmitter metabolism. Specific MAO inhibitors are employed for the management of depression and neurodegenerative disorders like Alzheimer’s and Parkinson’s disorders. Thus, a controlled breakdown of monoamines guarantees the apt working of neurotransmission at the synaptic cleft which is crucial for the regulation of the cerebral and other brain roles in the sensory system [22,23]. Presently, the U.S. Food and Drug Administration and European Medicines Agency approved drugs include donepezil, rivastigmine, tacrine, and galantamine. These medications are vital for the treatment of AD, though their efficacy is restricted because of their poor bioavailability, selectivity, and severe effects on the nervous system. Hence, the quest for new cholinesterase inhibitors and monoaminergic inhibitors is of importance [10,24,25,26]. 

Computational approaches such as quantitative structure-activity relationships (QSARs) have been effectively utilized to classify vital characteristics for specific inhibitory action. The QSAR is an approach to locate an association between the bioactivity of a compound and its fundamental chemical properties [27]. There are diverse relapse and design recognition methods that could be utilized for the variable determination and QSAR design [27]. The established procedures for QSAR are utilized predominantly in cheminformatics, drug discovery and to estimate the biological processes of unique biochemical principles, besides the pharmacokinetic assessments of specific chemicals [27,28]. There are several computational analyses documented for the detection of new components in the management of AD [29,30,31,32], but there is still no precise treatment for AD [29]. Previously, QSAR models have been developed for inhibitors targeting AChE, BChE and MAO enzymes. These models, though, were based on a 2-D QSAR and 3-D QSAR [33,34,35,36]. Islam et al. [37] developed a QSAR model utilizing quercetin as acetylcholinesterase enzyme inhibitor. Khatkar et al. [38] performed a QSAR model employing *p*-coumaric acid derivatives as AChE inhibitors. Mahmoodabadi et al. [39] developed a QSAR model with molecular dynamic studies using polyphenolic compounds as inhibitors of β-amyloid aggregation. Dhiman et al. [40] developed QSAR studies utilizing a large series of flavonoid derivatives (apigenin, kaempferol, luteolin and quercetin) as monoamine oxidase inhibitors. Das et al. [41] developed a QSAR model utilizing 21 known inhibitors of acetylcholinesterase. Mukesh and Dharmendra [42] reported a QSAR model for antioxidant and antimicrobial activity utilizing 18 flavonoid derivatives. Kondeva-Burdina et al. [43] reported a QSAR model utilizing nine flavonoids and two flavoalkaloids for hepto- and neuroprotective activity. Chakraborty et al. [44] developed a QSAR model utilizing a straight heuristic technique and built up a model utilizing 30 mixtures with defined BACE1 enzyme inhibitory action. In the current investigation, we have used a dataset of 74, 47 and 71 mixtures with AChE, BChE and MAO enzyme inhibitory properties for QSAR model development, utilizing simple meaningful and effectively interpretable descriptors. The created model is aimed toward giving measurable predictions for the AChE, BChE and MAO inhibitory properties of the compounds, expressed as the negative log of half maximal inhibitory activity (pIC_50_). Furthermore, [45] reported a QSAR model, molecular docking and simulation techniques for anti-Alzheimer’s agents. 

In this study, we developed an in silico auto-QSAR and pharmacophore model that can be utilized to screen the bioactivity of a dataset of nine compounds identified from *T. diversifolia*, *B. sapida,* and *I. gabonensis* that can repress the enzymatic functions of AChE, BChE, and MAO. Furthermore, we performed molecular docking studies as well as toxicity predictions with these active compounds.

## 2. Results

**Figure 1:**
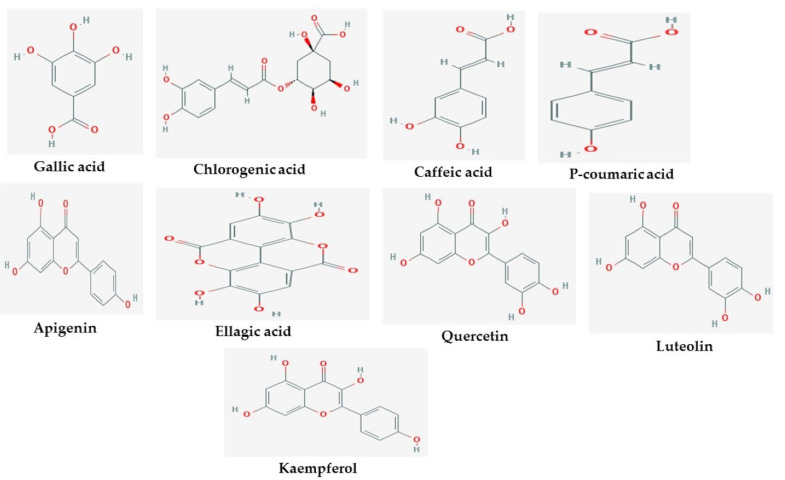
2D structures of studied ligands.

The 2D structures of the nine test compounds, namely gallic acid, chlorogenic acid, caffeic acid, *p*-coumaric acid, apigenin, ellagic acid, quercetin, luteolin, and kaempferol were modeled and used as a target for docking studies against three target proteins (AChE, BuChE and MAO) (Figure 1). They were selected based on compounds identified in the studied three medicinal plants from the literature.

A pharmacophore protocol which integrates the areas of structure-based and ligand-based procedures for AChE, BChE and MAO was explored in this study. Before energy-based site selection, the amount of pharmacophore sites for every ligand and the optimized hypothesis for AChE, BChE and MAO were determined (Table 1, Figures S1 and S2). Figure S1 reveals the last e-pharmacophores for every one of the nine ligands considered. The nine compounds generated 26 pharmacophore model hypotheses, each with a corresponding hypothesis score, the top ranked score being the best for each model.

These twenty-six models demonstrated three sorts of characteristics: A: Hydrogen Acceptor, D: Hydrogen Donor, and R: Aromatic ring. The hypothesis with six features was obtained from the crystal structure which indicated three aromatic rings (R) in contrast with different models (Table 1). The least featured pharmacophore with four points were acquired with four crystal structures (ADRR_1, AARR_1, AARR_2, AADR_1, ADRR_2, DRRR_1, ARRR_1, ADRR_3, and ARRR_2), among which ADRR_1, ADRR_2 and ADRR_3 revealed similar features, while ARRR_1 and AARR_1 had an additional ring and an additional acceptor individually instead of a hydrogen donor feature. The other five structures (AADRR_3, ADRRR_1, AADRR_1, AADRR_2, ADRRR_2, AARRR_1, DDRRR_1, ADRRR_3, AARRR_3 and AARRR_2) gave five-point pharmacophore hypotheses in which AADRR_3 and AADRR_2 displayed similar features, while ADRRR_1, ADRRR_2 and ADRRR_3 also showed similar features. In view of the most dynamically promising sites, we selected 4–6 features which were tried for performance for enhancing the active molecules utilizing a decoy set database. The enrichment results for all the targets utilizing the pharmacophore protocol method were contrasted for the enrichment factor (EF), BEDROC (α = 160.9), in light of the recovery rate of actives against the ranked decoys as shown in Table 1. The average EF 1% value from all 26 models was 36.76, while the average BEDROC value (α= 160.9) was ≥ 0.57. The e-pharmacophore method showed good enrichment of 1% (EF 1%) resulting in an EF (1%) of 36.76. The ROC plot (Figure S3) and % screen plot ROC plot (Figure S4) demonstrate that the used technique was subtle and precise in identifying the active compounds. Further, the ROC plots between percent screen and percent actives found were plotted and are portrayed in the Figure S3. These models are found to be sensitive and specific. Similarly, all the active compounds were identified early, signifying the robustness of the predicted models. The outcome shows that the models created from AChE, BChE and MAO could identify the actives, with a recovery rate of the actives found near 100% in the total ranked decoy database.

**Figure 2:**
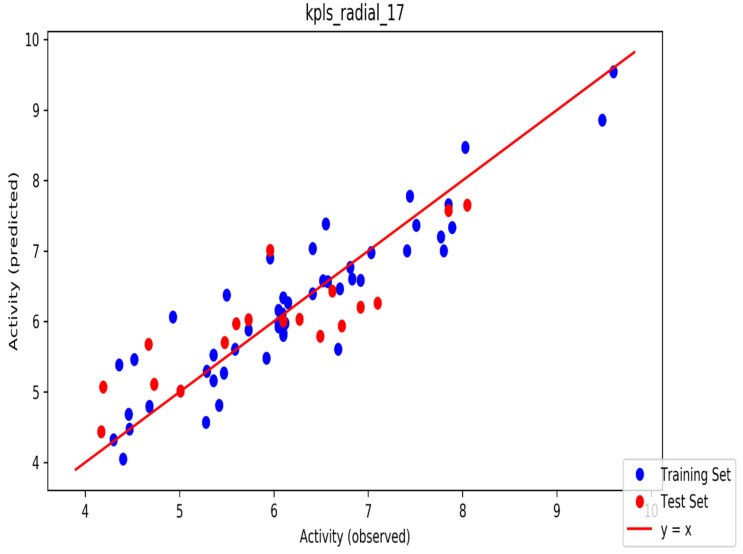
The scatter plot of observed and predicted values of the final partial least squares (PLS) model against the known AChE enzyme.

**Figure 3:**
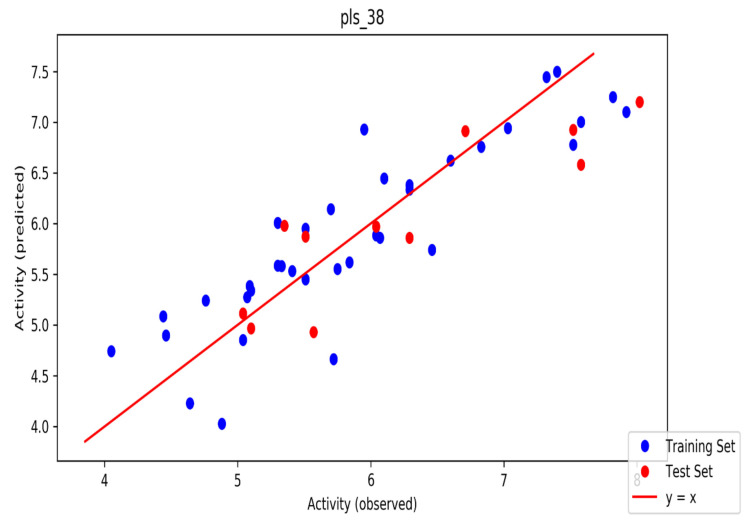
The scatter plot of observed and predicted values of the final PLS model against the known BChE enzyme.

**Figure 4:**
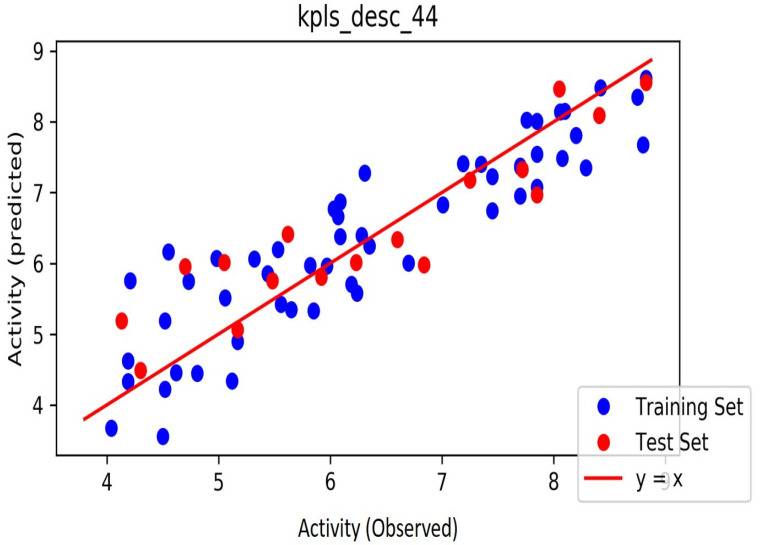
The scatter plot of observed and predicted values of the final PLS model against the known MAO enzyme.

The automated model divided the dataset arbitrarily into a 77% training set, and a 23% test set for AChE, BChE and MAO. Models are built on each training set from all possible combinations of machine learning methods, and sets of independent variables that are supported by each machine learning method. The algorithm created three best models and the outcomes are presented in Tables S1–S3. The pharmacophore models reveal moderately decent correlations between observed and predicted activities (Tables S1–S3) as indicated by the R^2^ and Q^2^ correlations. The best model kpls_radial_17 was recorded for AChE with a standard deviation (S.D) of 0.47, R^2^ of 0.86, root mean square error (RMSE) 0.58 and Q^2^ of 0.73 while the best model pls_38 recorded for BChE had a S.D of 0.51, R^2^ of 0.77, RMSE of 0.54, and Q^2^ of 0.72 and best model kpls_desc_44 recorded for MAO had a S.D of 0.64, R^2^ of 0.81, RMSE of 0.62, and Q^2^ of 0.81. The scatter plots showing predicted pIC_50_ versus observed pIC_50_ tests, respectively, for the best generated models are presented in Figure 2, Figure 3 and Figure 4. From the plots, it is observed that the QSAR model is able to predict the activity of training and test sets, respectively, expressed as the negative logarithm of the 50% inhibitor concentration (pIC_50_), to a large extent well; this is demonstrated in the fact that most points, especially in Figure 2 and Figure 4, are close to the regression line. The equations of the obtained models of QSAR analysis are as follows;

(1) $$Q2=1-\frac{PRESS}{SSY}=1-\frac{\Sigma \;(Yobs\left(training\right)-Ypred\;\left(training\right)x^{2}}{\;\Sigma \;(Yobs\left(training\right)-\bar{y}\left(training\right)x^{2}}$$

In the equation above, *ytraining* represents the average activity value of the training set, while *Yobs* (*training*) and *Ycal* (*training*) represent observed and predicted activity values, respectively, of training set compounds. Often, a high *Q2* value (*Q2 >* 0.5) is considered as a proof of the high predictive ability of the model:

(2) $$R2=1-\frac{\Sigma \;(Yobs\left(test\right)-Ypred\;\left(test\right)x^{2}}{\;\Sigma \;(Yobs\left(test\right)-\bar{Y}\left(training\right)x^{2}}$$

In the equation above, *Ypred* (*test*) and *Yobs* (*test*) signify the predicted and observed values, respectively, of the test set compounds and *ytraining* represents the mean activity value of the training set compounds. The value of *R2* pred for an acceptable model should be more than 0.5.

The binding affinity results of the ligands against the three selected targets of Alzheimer’s disease are presented in Table 2. The docking scores of the compound were between −6.10 and −10.40 kcal/mol. Luteolin achieved the highest binding affinity score of −10.40 kcal/mol, closely followed by apigenin and ellagic acid with docking scores of −10.20 and −9.80 kcal/mol for AChE, ellagic acid with a docking score of −9.90, luteolin and quercetin with scores of −9.70 and −9.60 kcal/mol for BChE and chlorogenic acid with a score of −9.90, followed by luteolin and ellagic acid with scores of −9.30 and −8.90 kcal/mol for MAO, respectively. The docking scores of donepezil and galanthamine were −10.70 and −7.5 kcal/mol for AChE, −9.70 and −8.60 for BChE and −10.80 and −6.10 for MAO, respectively. 

**Figure 5:**
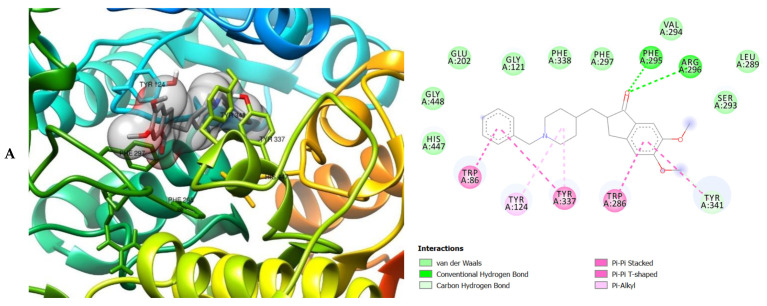

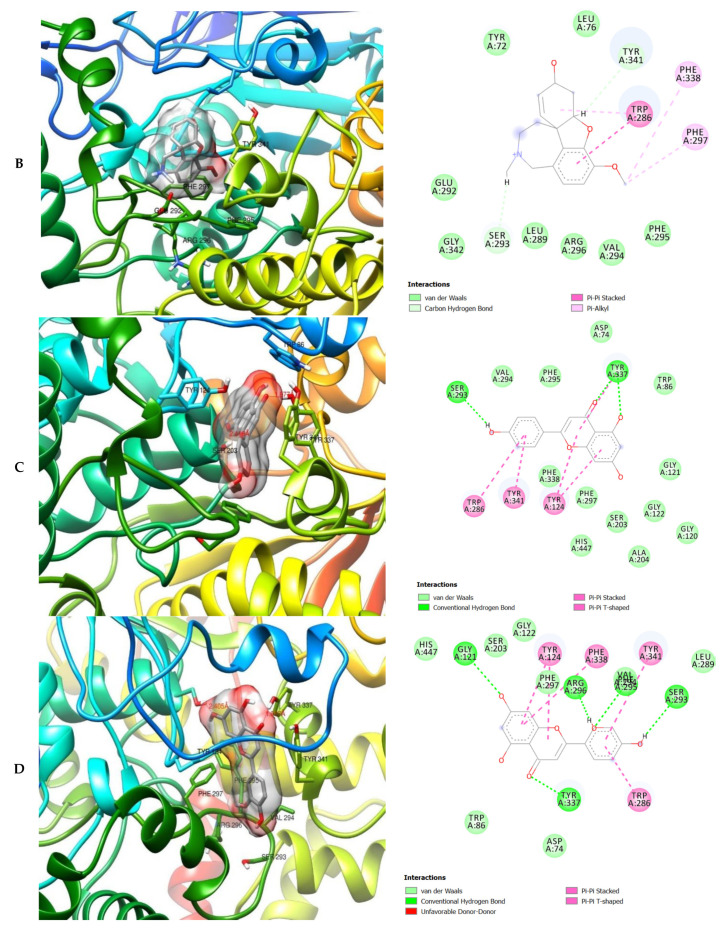

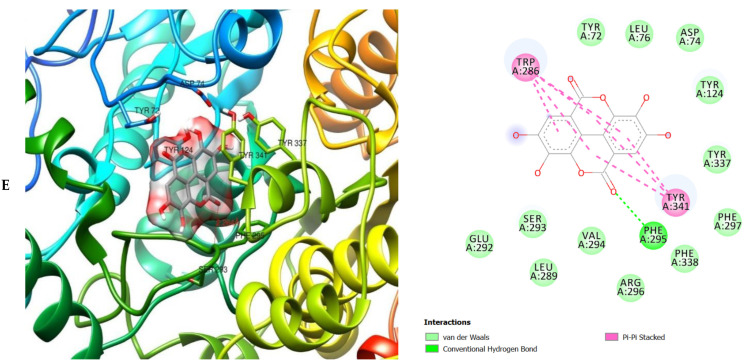
3D (left) and 2D (right) views of the molecular interactions of amino-acid residues of AChE (6U3P) with (**A**) donepezil, (**B**) galanthamine, (**C**) apigenin, (**D**) luteolin and (**E**) ellagic acid.

**Figure 6:**
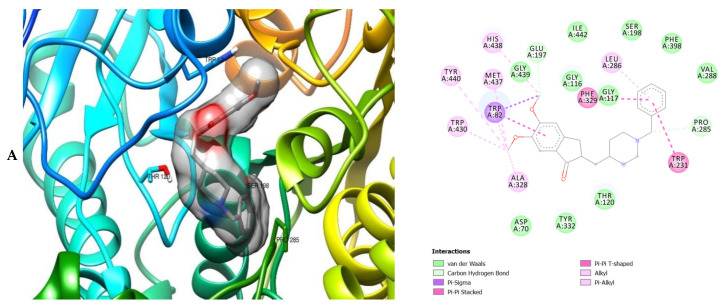

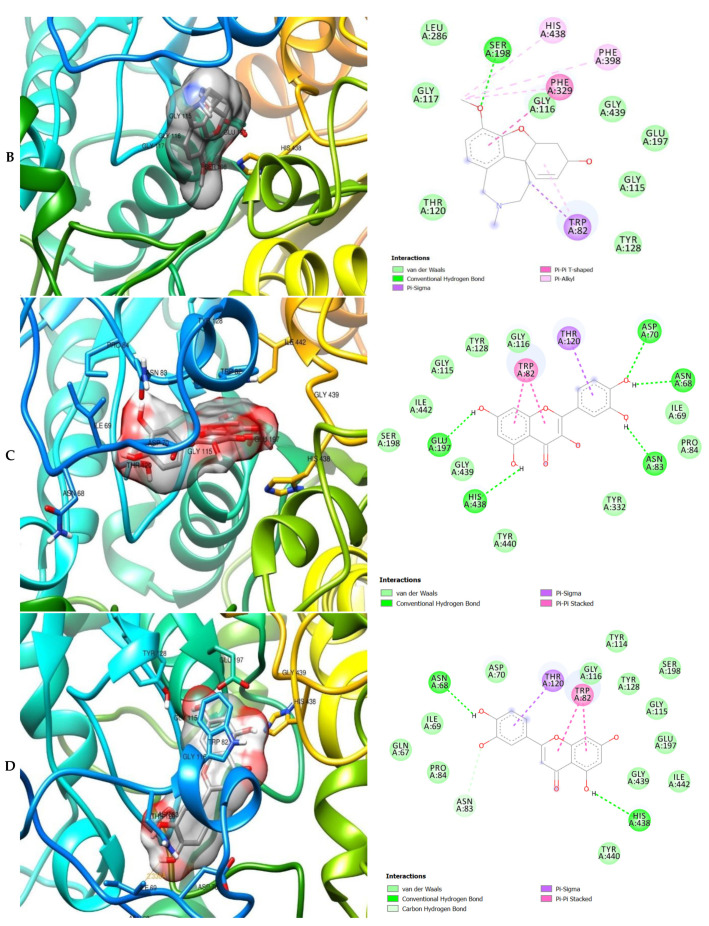

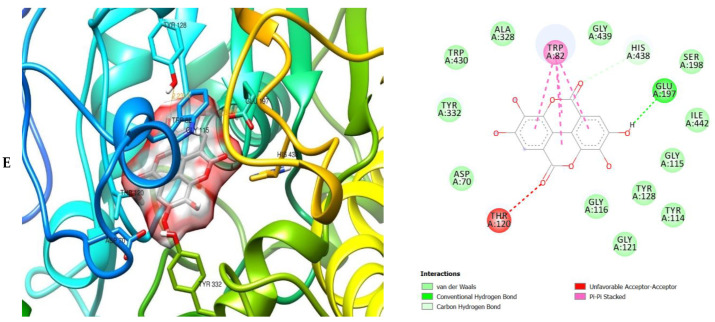
3D (left) and 2D (right) views of the molecular interactions of amino-acid residues of BChE (3O9M) with (**A**) donepezil, (**B**) galanthamine, (**C**) quercetin, (**D**) luteolin and (**E**) ellagic acid.

**Figure 7:**
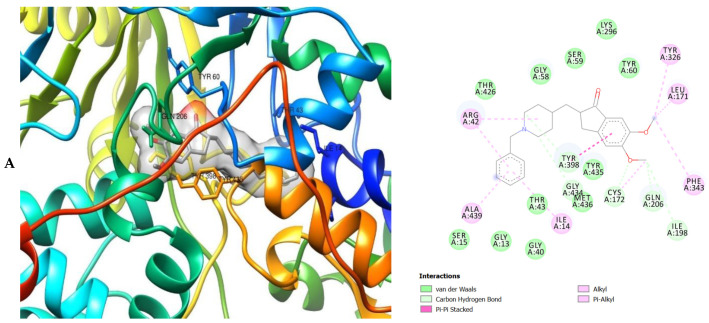

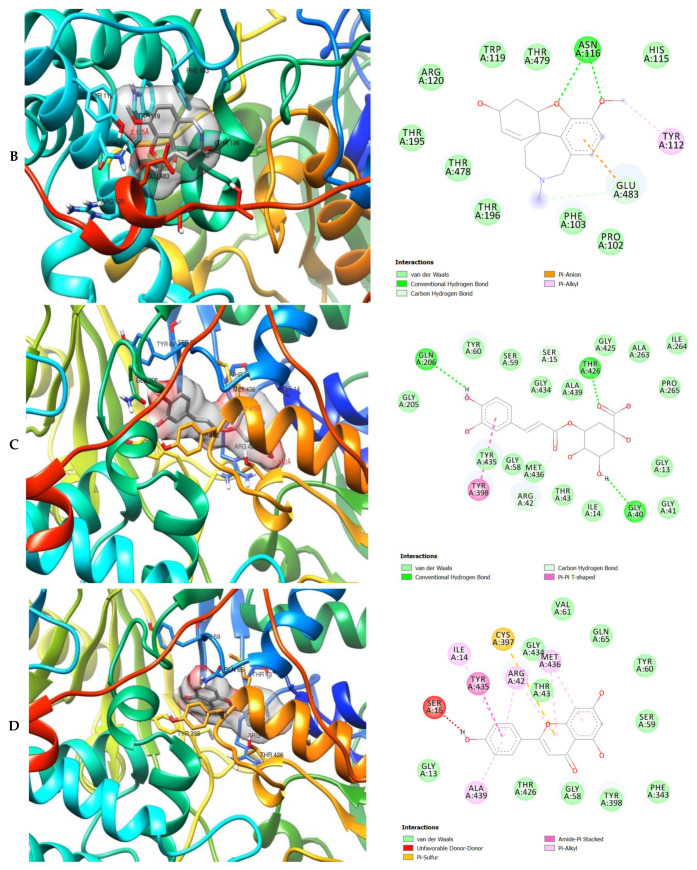

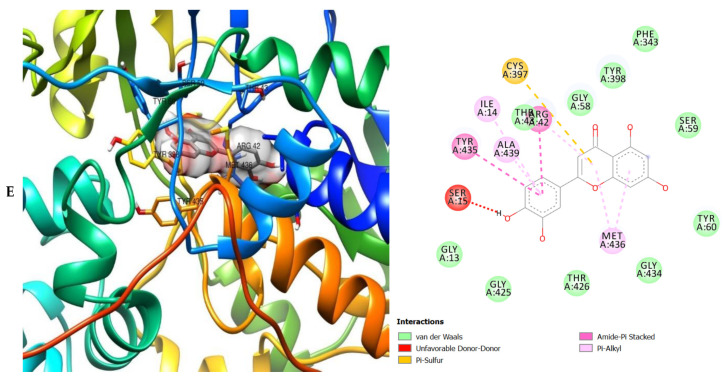
3D (left) and 2D (right) views of the molecular interactions of amino-acid residues of MAO (2BK5) with (**A**) donepezil, (**B**) galanthamine, (**C**) chlorogenic acid, (**D**) apigenin and (**E**) luteolin.

The molecular interactions of the amino acid residues of AChE, BChE, and MAO with the standards (donepezil and galanthamine), apigenin, luteolin, quercetin, chlorogenic acid and ellagic acid were determined and the results are presented in Figure 5, Figure 6 and Figure 7 The molecular docking study shows that the compounds interacted with several amino residues including HIS A:447, PHE A:338, PHE A:297, PHE A:295, VAL A:294, ARG A:296, SER A:293, GLU A:202, GLY A:448, GLY A:121, LEU A:289, ASP A:74, TRP A:86, PHE A:338, PHE A:297, HIS A:447, SER A:203, GLY A:121 and GLY A:122. The compounds interacted with the amino residues via numerous forces such as conventional hydrogen bonding, carbon-hydrogen bonds, π-interactions (e.g., π-alkyl bonds, π-sulfur, amide-π stacking, alkyl, π-π stacking and π-π-T-shaped stacking). 

The molecular bonding of the amino acid residues of acetylcholinesterase (AChE) with the standards (donepezil and galanthamine), apigenin, luteolin and ellagic acid are depicted in Figure 5A–E and Table S4. 

The molecular bonding of the amino residues of butyrylcholinesterase (BChE) with the standards (donepezil and galanthamine), quercetin, luteolin and ellagic acid are reported in Figure 6A–E and Table S5. 

The molecular interactions of the amino residues of monoamine oxidase (MAO) with the standards (donepezil and galanthamine), chlorogenic acid, apigenin and luteolin are represented in Figure 7A–E and Table S6. 

All nine ligand molecules identified from the three plants used were effectively docked against AChE, BChE and MAO, respectively. The molecules that had the most reduced binding energy were judged as the most excellent compounds in repressing the protein target as the reduced binding energy compares to greater affinity. Based on the nine ligand molecules, three ligands (apigenin, luteolin and ellagic acid) were chosen as the best ligands on the basis of their binding affinity against the selected targets (Table 2). 

Druglikeness prediction were performed for the test ligand molecules Lipinski’s RO5 shows the satisfactory scopes of the finest drug compound that are: molecular weight (MWt): ≤500, number of hydrogen bond donors: ≤5, number of hydrogen bond acceptors: ≤10, lipophilicity (expressed as LogP): ≤5 and molar refractivity (MR) from 40 to 130. All the nine ligands obeyed the RO5. Apigenin, luteolin, and ellagic acid have MWt ≤500 (270.24, 286.24, and 302.19 g/mol, respectively). The consensus logP values of apigenin, luteolin, and ellagic acid were 2.11, 1.73, and 1, respectively (Table 3). Furthermore, the MR of apigenin, luteolin, and ellagic acid were 73.99, 76.01, and 75.31, respectively. The values of LogS produced by apigenin, luteolin, and ellagic acid are −4.4, −3.82, and −3.35 (Table 4). Although, all of the compounds obeyed the Ghose, Veber, Egan and Muegge rules and revealed comparable bioavailability score of 0.55. Apigenin, luteolin, and ellagic acid produced synthetic accessibility (SA) scores of 2.96, 3.02, and 3.17, respectively. Furthermore, both apigenin and luteolin showed TPSA score of 90.90 and 111.13 Å. The rotatable bond for apigenin and luteolin are 1 and 4 while ellagic acid did not any. The standard drug donepezil and galantamine also revealed fairly worthy outcomes with no disobedience to all the rules. Donepezil and galantamine have MWt of 379.49 and 287.35 g/mol, respectively. 

All the compounds, except for chlorogenic acid, revealed high probabilities of being absorbed in the gastrointestinal tract (Table 5). The skin permeability (LogKp) is an important index for the evaluation of molecules that might require transdermal administration. The LogKp of the compounds is presented in Table 5. All the compounds are expected to be impermeable as they had the negative LogKp values. All ligand molecules revealed not to inhibit CYP2C9 and CYP2C19 whereas CYP1A2 and CYP2D6 was inhibited by apigenin, ellagic acid, kaempferol, luteolin, and quercetin (Table 5). The druglikeness properties of the compounds and the standards are presented in Table 6.

Pharmacokinetic tests were conducted on all the ligand molecules. Apigenin and luteolin showed Caco-2 permeability and all compounds were p-glycoprotein non-inhibitors (Table S7). Also, apigenin, luteolin and ellagic acid were p-glycoprotein substrates. All the ligands were found to be HIA positive, which means that they will be absorbed by the intestine. All the compounds did not show potential to cross the BBB which can be an advantage as they will have less likelihood to induce adverse effects in the sensory system. Two out of the three ligands were inhibitors for CYP450 1A2. However, only apigenin and luteolin were non-inhibitor for CYP3A4. Besides, all three compounds were non-substrates and non-inhibitors for CYP2C9 and CYP2C19. Apigenin, luteolin and ellagic acid all possess hepatotoxic properties (Table S8). Furthermore, none of the compounds were carcinogenic and mutagenic except for kaempferol. Donepezil and galantamine both revealed substrate properties to CYP2D6 and CYP3A4, while only donepezil showed inhibitory activity to CYP2D6. They were also substrates for CYP3A4. The results of ADME/T tests are listed in Tables S7 and S8.

## 3. Discussion

The pharmacophore models were created by PHASE [46,47,48,49] energetic terms onto pharmacophore sites that are calculated dependent on the structural and energy information between the protein and the ligand. The data obtained in this study are vital to evaluate the variety among the pharmacophore hypothesis based on different ligands. The utilization of various pharmacophore models created from various crystal structures cannot singly improve the possibility of recognizing molecules but also diversity and also the flexible nature of the active site can add to the modifications in the energy arrangement [50]. It is therefore very necessary to ascertain the screening approval of the pre-owned technique, if it was effective in the recovering of the actives from the records as well as placing them either early or not in an orderly manner by the pharmacophore model cycle. This study shows various authentications that were determined to recover the active compounds from the molecular records and it indicates that the screening procedures in recovering these records, were great [51]. Excluded volumes were accounted to better discern inactives and in doing that, different pharmacophore hypotheses were generated sorted on the values of the Phase Hypo Score, which is a linear combination of different contributes related with site, volume, vector and selectivity scores [46,52]. Enrichment factor (EF) was calculated as a point of reference for the reliability of the model and for the accurate ranking of compounds [53] For the hypothesis, EF1% value was 36.76 suggesting the superiority of pharmacophore modeling ranking over random. The enrichment results for all targets using the e-pharmacophore were compared for the enrichment factor (EF1%), BEDROC (a = 160.9), based on recovery rate of actives against the ranked decoy database as in Table 1. The average EF 1% value from all the hypothesis was >36.76 which is a good sign that this procedure can identify actives, while the average BEDROC values (a = 160.9) were ≥0.57. Another dependable metric to assess the performance of the pharmacophore hypothesis is the AUAC of the ROC curve (Table 1; Figures S3 and S4). The AUAC values of the models revealed comparable results as presented in Table 1. Further, the recovery rate of the known actives from the constructed decoy database versus the ranked database screened with 26 pharmacophore models were plotted and are depicted in the Figures S3 and S4. The result indicates that the pharmacophore models generated from the crystal structures of AChE, BChE and MAO could identify the actives, with a recovery rate of the known actives close to the total ranked decoy database. Based on the validation results these multiple pharmacophore hypotheses could be utilized for conscientiously recognizing potential hits. Based on this result it can be inferred that the model was better than a randomly generated model.

The bonding that takes place, which includes hydrogen bonds which interact like the carbon-hydrogen bond, conventional hydrogen bonds and π-interactions which includes π-sulphur, amide-π stacking, π-alkyl bonds, π-π stacking and π-π-T-shaped stacking. These occurs between the ligands and receptors which eventually played a significant part in the probably played a significant role in the repressive activity on the enzymes as well as attractive charge [54,55]. Therefore, it can be affirmed that hydrophobicity, electrostatic interactions, hydrogen bonding effect, and unsaturation features (which are mediated by π-interactions) which was revealed in the docking study as well as the auto-QSAR modeling and are necessary for the repressive performance against AChE, BChE and MAO. Validation methods are necessary to establish the robustness of a model on unseen data. The method of root mean-squared error (RMSE) is one of the internal methods of validating a model [56]. The screening process additionally progressed via the use of the machine learning-based predictive model (pIC_50_ calculation) performed by the Auto-QSAR panel of Schrodinger. Thus, given a learning set of chemical structures and an activity property from CHEMBL database, a total of 497 physicochemical and topological descriptors were computed, together with a variety of Canvas fingerprints [52], providing a large pool of independent variables from which to build models.

The strategies for external validation are crucial and it is of paramount interest to adopt all available validation strategies to check robustness of the model. The parameters for external validation such as Q^2^ and R^2^ were used in the QSAR model report in this study. The auto-QSAR model for AChE, BChE and MAO had R^2^ of 0.86, 0.77 and 0.81 for the training set of compounds with Q^2^ values of 0.73, 0.72 and 0.81 for test set of compounds. The slopes of regression line and correlation coefficient were obtained from predicted pIC_50_ and observed pIC_50_ activity of the dataset. All the parameters for external validation of pharmacophore models like cross validation (Q2) values, the correlation coefficient (R2) values, for AChE, BChE and MAO indicated that the model had high predictive ability. The calculated pIC50 values of the compounds in the predicted test set and observed test set are listed in Tables S1–S3. These scatter plots are significant for the predictive ability of QSAR. Residual plots (scatter) were utilized to identify the existence of outliers from a QSAR model [57,58]. Thus, the developed QSAR model was considered stable and as expected, it was able to validate the observed pIC50 values for the compounds. A predictive correlation coefficient R^2^ values of 0.86, 0.77, 0.81 for the set were achieved for the developed model. In general, statistical values of R^2^ >0.6 and Q^2^ >0.5 between the predicted and the observed values portrays the model to be good and able to predict the AChE, BChE and MAO inhibitory activities of compounds not included in the model development process [57,58,59].

The current survey made use of Autodock vina and flexible docking to correctly predict the binding affinity and docking score of the compounds derived from *T. diversifolia*, *B. sapida,* and *I. gabonensis* respectively with AChE, BChE and MAO, as such denoting compounds with favorable interaction. These therefore suggest that the compounds derived from *T. diversifolia*, *B. sapida*, and *I. gabonensis* possess some bioactivities against AD.

As can be seen from the molecular docking results, the least active compound was galanthamine, it interacted with the least number of amino acid residues of AChE, BChE and MAO whereas the most active were donepezil, quercetin and chlorogenic acid (Figure 5, Figure 6 and Figure 7). All the compounds interacted strongly with the amino acid residues of AChE, however apigenin was the most active as it interacted with the highest number of amino acid residues of AChE and with the highest number of interacting forces. This suggests that apigenin possess the most important inhibitory activity against AChE and therefore can serve as a potential acetylcholinesterase inhibitor (AChEI). Donepezil shows the most bioactivity against BChE. The compound interacted with the highest number of amino acid residues of BChE, with the highest number of interacting forces (seven different interactions) in comparison to the other compounds. This was closely followed by quercetin which interacted with twenty-two amino acid residues mediated by four interacting forces. It therefore can be proposed that donepezil and quercetin possess higher bioactivity against BChE. The most active compounds against MAO were donepezil and chlorogenic acid when compared with the other compounds indicating their preference as inhibitors of MAO due to their higher level of bioactivity (interaction with the amino acid residues of MAO) against the enzyme. They reacted with twenty-two amino acid residues of MAO each. Donepezil interacted with five different interacting forces whereas chlorogenic acid was four.

In this study, the molecular docking approach was conducted to examine the most favorable position by a ligand molecule inside the binding pocket of a limited receptor after which a binding power is determined. The lesser the binding vigor, the higher the chances of binding and vice-versa. In the present study, nine ligand molecules identified from *T. diversifolia*, *B. sapida*, and *I. gabonensis*, respectively, were analyzed to inhibit the AChE, BChE and MAO enzyme that are responsible for AD progression. Each of the nine ligands were docked against the selected target receptor to determine their anti-Alzheimer’s activity and from the experiments, the three best ligands were selected for further analysis. The best possible ligand compounds were selected based on their binding energy, where the lower bind energy was favored.

The moment the three terrific ligands were placed side by side with the positive control, the performance of donepezil was noticed to be satisfactory from the docking studies, while apigenin, luteolin and ellagic acid was observed to be more satisfactory than galantamine. Therefore, it can then be stated conclusively, that the larger performances that was revealed in this study was that of the three terrific ligand molecules. Figure 6 shows the amino acids that took part in the cooperation between the ligands and the positive controls with AChE. PHE the most frequently interacting amino acid that was observed among all amino acids of AChE coupled with the three ligands as well as positive controls (donepezil and galantamine) is PHE A: 338 amino acids. There was a prediction that all three ligands will interact with PHE A-338, they all also interacted with TRP A:341, TRP A:286 and PHE A:295 by hydrogen and hydrophobic interactions. Furthermore, interactions of the three ligands with BChE revealed amino acids with TRP A:440, TRP A:8 and HIS A:438 via hydrogen and hydrophobic interactions, while interactions with MAO by hydrogen and hydrophobic interactions showed amino acids with TYR A:398 and ASN A:116. It could then be stated conclusively, that the binding that takes place between the active sites of AChE, BChE and MAO receptor and all the three ligands were very vital for receptor-ligand interactions and also for strengthening, because of the hydrogen and hydrophobic interactions [60,61,62].

Assessing the druglikeness enables the processes of discovering drug and producing them. For a drug to penetrate through the biological barrier, the topological polar surface area (TPSA) and the molecular weight must be considered. The higher the TPSA and molecular weight values, the less the drug candidate is able to penetrate and vice versa. The partition coefficient logarithm of a drug compound in an organic or liquid phase (LogP) is termed lipophilicity. It influences the digestion of the drug compounds in the body and increased LogP implies decreased digestion and vice-versa. The ability of a drug compound to dissolve is impacted by its LogS value and the least value is better. In addition, the ability of a drug molecule to penetrate the cell layer is influenced by the amount of donors and acceptors of hydrogen bond it possesses above the required ranges. Rotatable bonds number influences the properties of druglikeness and the range for the acceptable number is <10. In addition, the Lipinski’s RO5 shows that an effective drug molecule ought to include properties within the acceptable range of the five Lipinski guidelines [63]. The druglikeness prediction was performed for all the ligand molecules. Also, as per a potential drug molecule like Ghose filter which is supposed to have an estimated LogP of −0.4–5.6, and molecular weight of about 160 and 480, an absolute number of 20 to 70 atoms, and about 40 to 130 molar refractivity, which is suitable as an effective drug [64]. Oral bioavailability of a potential drug compounds depends on two aspects according to Veber rule and they include; Polar surface that ought to be equivalent to 140 Å2 and 10 or lower rotatable bonds numbers [65]. Moreover, as per the Egan rule, the absorption of a potential drug compound additionally relies upon two elements: the polar surface area (PSA) and AlogP98 (the logarithm of partition co-efficient between n-octanol and water) [66]. Furthermore, as per the Muegge rule, for a drug-like substance to become an effective compound, it must go through a filter that was created by researchers which is known as the pharmacophore point filter [67]. Furthermore, the synthetic accessibility (SA) score will be used to assess a target molecule before it is synthesized. There are different scores and the syntheses vary on the score for example score 10 means very hard and difficult to synthesize and score 1 simply means very easy and simple to synthesize [68]. The bioavailability score determines the penetrability of a potential drug molecule as well as the bioavailability properties [69]. 

According to the respective molecular weights of apigenin (270.24 g/mol), luteolin (286.24 g/mol) and ellagic acid (302.19 g/mol) apigenin ought to be the best one among the three ligands because a lesser molecular weight is always significant. The TPSA values of apigenin and luteolin revealed values of 90.90 and 111.13, respectively. Since, great outcomes have consistently resulted from a lesser TPSA value, apigenin should perform better than luteolin and ellagic acid. On account of the lipophilicity (LogP), a lower value is consistently essential. The least logP value corresponded to ellagic acid, among all three ligands, which exhibited an outstanding performance in the lipophilicity study. The remaining two ligands (apigenin and luteolin) also exhibited outstanding performance as well, having logP values of 2.11 and 1.73, respectively, additionally demonstrating very good performances. Apigenin, luteolin and ellagic acid were all predicted to obey the five Lipinski rules. It was further observed that the Ghose filter, Muegge, Veber, and Egan rules were obeyed by the three ligands too. Looking closely at all the phases of the druglikeness prediction study, it stands to reason that the compounds apigenin, luteolin and ellagic acid (the three best ligands) did comparatively well in the druglikeness prediction study and all three ligands demonstrated very stable actions in the druglikeness prediction study when compared side by side with the controls.

All the compounds, except for chlorogenic acid, showed high probabilities of being absorbed in the gastrointestinal tract (GIT). This suggests that these compounds have the potential to be absorbed in the GIT upon oral administration [70]. Metabolism prediction of principal compounds is one of the main concerns in the course of drug discovery [71]. The metabolism predictions of the compounds were achieved against five isoforms of cytochrome P450 (CYP) monooxygenase family namely; CYP1A2, CYP2C19, CYP2C9, CYP2D6 and CYP3A4. Cytochrome P450 monooxygenase performs a vital function in the drug metabolism and elimination process. The non-inhibition action of the identified compounds against these enzymes indicates that the compounds have high probabilities of been transformed and consequently be bioavailable upon oral administration [71]. Alternatively, the inhibition of the CYP isomers by the compounds can lead to poor bioavailability owing to failure to be metabolized and toxic side effects attributable to their accumulation [72]. The skin is a selective barrier that allows diverse compounds to permeate through at diverse rates based on their physicochemical properties [46]. Hence, the skin permeability (LogKp) is a key parameter for the evaluation of molecules that might require transdermal administration. All the molecules are expected to be impermeable as they had negative LogKp values. This implies that none of the molecules could be effectively administered through the skin [73].

ADMET predictions were determined to assess the potential of a drug molecule within a biological system from its pharmacological and pharmacodynamic properties. Thus, the achievement of a drug investigation and drug improvement is a vital factor, while the main factor for the drugs that principally focus on the brain cells is the blood brain barrier (BBB). Besides, inasmuch as a large portion of the drugs are administered orally, it is of great importance that the intestinal tissue digests the drug compounds. A well-characterized plasma *membrane* ATP-binding cassette *transporter*, known as *P*-*glycoprotein* (*P*-*gp*), *helps in transporting drugs*, and by so doing prevents p-gp and influences the drug transportation. Penetrability studies through in-vitro investigation generally utilize the cell line called Caco-2. The penetrability of the drug determines if the intestine will effectively digest the drug molecule or not. Orally administered drugs normally return back to the liver after making their journey through the bloodstream. An enzyme of cytochrome P450 family, uses the drugs as substrate and finally ejects the drugs through the urine or bile. Consequently, interference of any kind with any of these enzymes leads to a breakdown of the drug molecule [49,74]. Additionally, when a compound is discovered to be a substrate for at least one type of CYP450 enzyme, it means the compound is anticipated to be easily and seriously metabolized by the respective CYP450 enzyme or enzymes [19]. Another influencer of the pharmacodynamics, circulation and excretion of the drug is the ability of a drug to bind to plasma proteins which also signifies a pharmacological index. The level at which a drug binds to plasma proteins actually determines its capability. Orally managed drugs have a significant cycle which is known as human intestinal absorption (HIA), which shows the digestion of drugs taken through the mouth down to the intestine and into the circulatory system [75]. The human liver is the primary site where metabolism take place could be defenseless against the impacts of toxic agents and various drugs. Human hepatotoxicity (H-HT) demonstrates and shows various kind of harm to the liver that could cause the organ to fail or eventually lead to death [74]. There is a mutagenicity test called the Ames test that is used to recognize compounds that have a potential to be mutagenic causing alterations or malignant growth [47]. In the digestion area, there was a flawless performance by the ligands. However, apigenin and luteolin which are non-inhibitors of p-gp display Caco-2 permeability Consequently, none of the tested plant natural products stopped or prevented the actions that are made possible by p-gp. In any case, as a result apigenin, luteolin and ellagic acid were p-gp substrates, and this enables them to be absorbed more effectively by cells. Again, the human intestine will be able to digest the HIA capability, as the three ligands have shown us. In the metabolism area, apigenin, luteolin and ellagic acid were inhibitors of CYP1A2 while apigenin and luteolin are inhibitors of CYP3A4 suggesting very effortless metabolism of the ligands. In the toxicity area, all three ligands were tested in the Ames mutagenicity and carcinogenicity tests and shown to be safe. Moreover, none was human hepatotoxic when placed side by side in comparison with the two controls that were used in this study. Therefore, in this study, apigenin, luteolin and ellagic acid displayed excellent performance in comparison with the positive controls in some areas of the study. Placed side by side with the two controls, it could be conclusively said that apigenin, luteolin and ellagic acid demonstrated acceptable outcomes in the druglikeness, molecular docking study, and ADMET prediction. 

## 4. Materials and Methods

### 4.1. Proteins and Ligands Collection

Nine test compounds, namely gallic acid, chlorogenic acid, caffeic acid, p-coumaric acid, apigenin, ellagic acid, quercetin, luteolin, and kaempferol identified from *T. diversifolia*, *B. sapida,* and *I. gabonensis*, respectively, were subjected to molecular docking analysis against the three AD protein biomarkers targeted in this study: acetylcholinesterase (PDB ID: 6U3P), butyrylcholinesterase (PDB ID: 3O9M), and monoamine oxidases (PDB ID: 2BK5). The crystal structures of Alzheimer’s protein biomarkers were obtained from the Protein Data Bank (PDB) and the structure data file (SDF) format of the test compounds was obtained from the PubChem database. PDBQT format of the PDB and SDF files for target-ligand docking were prepared using AutoDock 4.2. Finally, the results were analyzed using UCSF Chimera 1.14 and Discovery Studio 2020 [75,76].

### 4.2. Generation of Ligand-Based Pharmacophore Model

The structure data file (sdf) of our test compounds sourced from PUBCHEM database were prepared using LigPrep panel of the Schrödinger suite (Schrödinger 2020-3, LLC, New York, NY, USA), the chemistry of the ligands was properly standardized and extrapolated, and were used for the pharmacophore modeling using PHASE.

The ligands were automatically aligned by PHASE based on their best arrangement and mutual features. To develop the pharmacophore model, the prepared ligands were imported to the maestro workspace, and based on their experimental binding affinities (pIC50), the ligands were defined as active or inactive. [pIC_50_ = −log(IC_50_)]. IC_50_ ≤ 50 nM affinity corresponds to a pIC_50_ ≥ 7.3. The threshold for recognizing an inactive molecule is 10 μM or pIC_50_ ≤ 5.0.

The hypothesis requirement was set to match 50% of the actives, Number of features in the hypothesis set to five as the preferred minimum number of features to match. The hypothesis difference criteria were left as the defaults except for the donor and negative ionic features that were set to 1, hence, the acceptor and negative features were made equivalent [77]. 

### 4.3. Machine Learning Development of Automated QSAR Model

#### Dataset Generation and Preparation

The experimental dataset containing ACHE, BCHE and MAO inhibitors were recovered from the CHEMBL database (www.ebi.ac.uk/chembl/) (accessed on 20 October 2020), through blasting of the FASTA sequence of the particular proteins. Bioactive inhibitors of the proteins were retrieved with their respective pIC_50_ values from CHEMBL. The bioactive inhibitors with pIC_50_ were converted to structure data file (sdf) format utilizing the Data-Warrior package (v.2) [78]. The sdf format was transferred to the working area of Maestro for preparation by ligprep [79]. The prepared compounds were transferred to the Canvas cheminformatics program [80] for clustering base on the Tanimoto similarity between sets of hashed linear binary fingerprint descriptors, and to decide the primary structural variety among the inhibitor, thus, to choose representatives from each resulting cluster. These computational studies generated a total of 74, 47 and 71 clusters and were used to build the QSAR model using the automated QSAR panel of Maestro Schrödinger Suite.

The QSAR models of each target proteins were built based on the IC_50_ of the corresponding ligands. For AChE, kpl_radial_17 was the best model chosen based on the prediction ranking of the all-model outcomes; BChE, pls_38 and MAO, kpls_desc_44 was chosen respectively. Furthermore, the predictive precision of the model is assessed utilizing different indices like ranking score, root mean square error (RMSE), standard deviation (SD), Q^2^ and R^2^ values [81]. Furthermore, the predictive capability of a QSAR model can be assessed by the accompanying statistical attributes of the test set which was suggested by [57]: namely the correlation coefficient *R* between the predicted and observed activities.

### 4.4. Virtual Screening

Autodock vina in the PyRx software was utilized to achieve the docking based virtual screening of nine candidate compounds against the target protein receptors. The structure data files (sdf) of the candidate compounds were obtained from PubChem. The candidate compounds were subjected to molecular docking utilizing the AutoDock Vina program in the PyRX software. The ligands with high docking scores were filtered by Lipinski’s rule and by their SwissADME physicochemical properties. Only the molecules which followed Lipinski’s rules and SwissADME predictions were considered as hits and subjected to post-docking analysis using Discovery Studio 2020 and UCSF Chimera 1.14, and toxicity screening using the admetSAR web server. Hence, the top-ranked compounds were suggested for experimental screening for the establishment of therapeutic interventions against Alzheimer’s [76].

### 4.5. ADMET Screening

Absorption, distribution, metabolism, excretion, and toxicity (ADMET) of the test compounds were determined utilizing an in silico integrative model predicted at the SwissADME and admetSAR web servers, respectively. Using a huge database, these servers speculate the physicochemical properties, pharmacokinetics, water- dissolvability, lipophilicity, drug-likeness, therapeutic properties, and toxicity of compounds with high precision [82].

## 5. Conclusions

This research work provides valuable evidence on the appropriate nature of novel AChE/BuChE and MAO inhibitors as potential anti-AD candidates. We conclude that the compounds from this study demonstrate potential neuroprotective property by virtue of binding to the key protein targets for Alzheimer’s. Pharmacophore modeling and Auto-QSAR models were performed with a good correlation coefficient, which brought about predicting the inhibitory activities for AChE, BChE and MAO. Luteolin revealed better absorption and BBB permeability than other compounds, which indicates it could be a potential candidate for AD treatment. Luetolin showed the most binding affinity against AChE/BuChE and MAO followed by apigenin and ellagic acid while the least was presented by gallic acid. In all, luteolin was revealed as the most active flavonoid of all the compounds tested.

## Figures and Tables

**Table 1:** Statistic variations of the pharmacophore model.

ID	Phase Hypo Score	EF1%	BEDROC160.9	ROC	AUAC	Average Outranking Decoys	Total Actives	Ranked Actives	Matches	Excluded Volumes
ADRR_1	0.83	36.76	0.58	0.45	0.69	3.2	11	5	4 of 4	No
AARR_1	0.82	36.76	0.58	0.45	0.66	3.4	11	5	4 of 4	No
AADRR_3	0.81	36.76	0.58	0.45	0.7	3	11	5	5 of 5	No
AARR_2	0.81	36.76	0.57	0.45	0.64	5.6	11	5	4 of 4	No
AADRRR_1	0.8	36.76	0.57	0.36	0.67	0	11	4	6 of 6	No
ADDRRR_1	0.8	36.76	0.57	0.36	0.68	0	11	4	6 of 6	No
AAARRR_1	0.8	36.76	0.57	0.36	0.67	0	11	4	6 of 6	No
AADRRR_2	0.79	36.76	0.57	0.36	0.68	0	11	4	6 of 6	No
AAADRR_1	0.79	36.76	0.57	0.36	0.66	0	11	4	6 of 6	No
ADRRR_1	0.78	36.76	0.57	0.36	0.66	0	11	4	5 of 5	No
AAADRR_2	0.78	36.76	0.57	0.36	0.66	0	11	4	6 of 6	No
AADDRR_1	0.78	36.76	0.57	0.36	0.67	0	11	4	6 of 6	No
AADRR_1	0.77	36.76	0.57	0.36	0.63	0	11	4	5 of 5	No
AADRR_2	0.77	36.76	0.57	0.36	0.64	0	11	4	5 of 5	No
ADRRR_2	0.77	36.76	0.57	0.36	0.64	0	11	4	5 of 5	No
AARRR_1	0.77	36.76	0.57	0.36	0.64	0	11	4	5 of 5	No
DDRRR_1	0.77	36.76	0.57	0.36	0.67	0	11	4	5 of 5	No
AARRR_2	0.77	36.76	0.57	0.36	0.63	0	11	4	5 of 5	No
AADR_1	0.77	36.76	0.57	0.45	0.66	15.6	11	5	4 of 4	No
ADRRR_3	0.77	36.76	0.57	0.36	0.66	0	11	4	5 of 5	No
AARRR_3	0.77	36.76	0.57	0.36	0.64	0	11	4	5 of 5	No
ADRR_2	0.76	36.76	0.57	0.36	0.61	0	11	4	4 of 4	No
DRRR_1	0.76	36.76	0.57	0.36	0.61	0	11	4	4 of 4	No
ARRR_1	0.75	36.76	0.57	0.36	0.59	0	11	4	4 of 4	No
ADRR_3	0.75	36.76	0.57	0.36	0.55	0	11	4	4 of 4	No
ARRR_2	0.75	36.76	0.57	0.36	0.59	0	11	4	4 of 4	No
ARRR_3	0.75	36.76	0.57	0.36	0.59	0	11	4	4 of 4	No

Legend: EF1% = Enrichment factor; BEDROC160.9 = Boltzmann-enhanced discrimination of receiver operating characteristic; ROC = Receiver operating characteristic curve; AUAC = Area under accumulation curve; A = hydrogen bond acceptor; D = hydrogen bond donor; R = Aromatic ring.

**Table 2:** Binding affinity (kcal/mol) of test compounds against selected Anti-Alzheimer’s target.

Compounds	6u3p_AChE	3o9m_BChE	2bk5_MAO
**Apigenin**	−10.2	−9.4	−9.2
**Caffeic Acid**	−7.2	−6.7	−7.8
**Chlorogenic acid**	−9.6	−8.6	−9.9
**(*R*)-Donepezil**	−10.7	−9.7	−10.8
**Ellagic acid**	−9.8	−9.9	−8.9
**Galantamine**	−7.5	−8.6	−6.1
**Gallic acid**	−6.5	−6.1	−6.3
**Kaempferol**	−9.6	−9.4	−8.4
**Luteolin**	−10.4	−9.7	−9.3
***p*-Coumaric acid**	−7.1	−6.6	−7
**Quercetin**	−9.4	−9.6	−8.8

**Table 3:** Predicted lipophilicity (Log P) values.

Compounds	iLOGP	XLOGP3	WLOGP	MLOGP	Silicos-IT Log P	Consensus Log P
Apigenin	1.89	3.02	2.58	0.52	2.52	2.11
Caffeic Acid	0.97	1.15	1.09	0.7	0.75	0.93
Chlorogenic acid	0.87	−0.42	−0.75	−1.05	−0.61	−0.39
(*R*)-Donepezil	3.92	4.28	3.83	3.06	4.91	4
Ellagic acid	0.79	1.1	1.31	0.14	1.67	1
Galanthamine	2.67	1.84	1.32	1.74	2.03	1.92
Gallic acid	0.21	0.7	0.5	−0.16	−0.2	0.21
Kaempferol	1.7	1.9	2.28	−0.03	2.03	1.58
Luteolin	1.86	2.53	2.28	−0.03	2.03	1.73
*p*-Coumaric acid	0.95	1.46	1.38	1.28	1.22	1.26
Quercetin	1.63	1.54	1.99	−0.56	1.54	1.23

**Table 4:** SwissADME predicted bioavailability and water solubility (Log S) values of test compounds.

Compounds	ESOL Log S	ESOL Solubility (mg/mL)	ESOL Class	Ali Log S	Ali Solubility (mg/mL)	Ali Class	Silicos-IT LogSw	Silicos-IT Solubility (mg/mL)	Silicos-IT Class	Bio-Availability Score
Apigenin	−3.94	3.07 × 10^−2^	Soluble	−4.59	6.88 × 10^−3^	Moderately soluble	−4.4	1.07 × 10^−2^	Moderately soluble	0.55
Caffeic Acid	−1.89	2.32 × 10^0^	Very soluble	−2.38	7.55 × 10^−1^	Soluble	−0.71	3.51 × 10^1^	Soluble	0.55
Chlorogenic acid	−1.62	8.50 × 10^0^	Very soluble	−2.58	9.42 × 10^−1^	Soluble	0.4	8.94 × 10^2^	Soluble	0.55
(*R*)- Donepezil	−1.481	5.87 × 10^−3^	Soluble	−4.81	5.92 × 10^−3^	Moderately soluble	−6.9	4.78 × 10^−5^	Poorly soluble	0.56
Ellagic acid	−2.94	3.43 × 10^−1^	Soluble	−3.66	6.60 × 10^−2^	Soluble	−3.35	1.36 × 10^−1^	soluble	0.55
Galanthamine	−2.93	3.41 × 10^−1^	Soluble	−2.34	1.31 × 10^0^	Soluble	−2.96	3.17 × 10^−1^	soluble	0.55
Gallic acid	−1.64	3.90 × 10^0^	Very soluble	−2.34	7.86 × 10^−1^	Soluble	−0.04	1.55 × 10^2^	Soluble	0.56
Kaempferol	−3.31	1.40 × 10^−1^	Soluble	−3.86	3.98 × 10^−2^	soluble	−3.82	4.29 × 10^−2^	Soluble	0.55
Luteolin	−3.71	5.63 × 10^−2^	Soluble	−4.51	8.84 × 10^−3^	Moderately soluble	−3.82	4.29 × 10^−2^	Soluble	0.55
*p*-Coumaric acid	−2.02	1.58 × 10^0^	Soluble	−2.27	8.73 × 10^−1^	Soluble	−1.28	8.67 × 10^0^	Soluble	0.56
Quercetin	−3.16	2.11 × 10^−1^	Soluble	−3.91	3.74 × 10^−2^	Soluble	−3.24	1.73 × 10^−1^	soluble	0.55

**Table 5:** Pharmacokinetics prediction output of test compounds.

	Apigenin	Caffeic Acid	Chlorogenic Acid	(*R*)-Donepezil	Ellagic Acid	Galanthamine	Gallic Acid	Kaempferol	Luteolin	*p*-Coumaric Acid	Quercetin
GI absorption	High	High	Low	High	High	High	High	High	High	High	High
BBB permeant	No	No	No	Yes	Yes	No	No	No	No	Yes	No
Pgp substrate	No	No	No	Yes	Yes	No	No	Yes	Yes	No	Yes
CYP1A2 inhibitor	Yes	No	No	No	Yes	No	No	Yes	Yes	No	Yes
CYP2C19 inhibitor	No	No	No	No	No	No	No	No	No	No	No
CYP2C9 inhibitor	No	No	No	No	No	No	No	No	No	No	No
CYP2D6 inhibitor	Yes	No	No	Yes	No	Yes	No	Yes	Yes	No	Yes
CYP3A4	Yes	No	No	Yes	No	No	Yes	Yes	Yes	No	Yes
Skin permeability logKp (cm/s)	−5.8	−6.58	−8.76	−5.58	−7.36	−6.75	−6.84	−6.7	−6.25	−6.26	−7.05

**Table 6:** Druglikeness prediction output of test compounds.

	Apigenin	Caffeic Acid	Chlorogenic Acid	(*R*)- Donepezil	Ellagic Acid	Galanthamine	Gallic Acid	Kaempferol	Luteolin	*p*-Coumaric Acid	Quercetin
MW	270.24	180.16	354.31	379.49	302.19	287.35	170.12	286.24	286.24	164.16	302.24
#Heavy atoms	20	13	25	28	22	21	12	21	21	12	22
#Aromatic heavy atoms	16	6	6	12	16	6	6	16	16	6	16
Fraction Csp3	0	0	0.38	0.46	0	0.53	0	0	0	0	0
#Rotatable bonds	1	2	5	6	0	4	1	4	4	4	2
#H-bond donors	3	3	6	0	4	1	4	4	4	2	5
MR	73.99	47.16	83.5	115.31	75.31	84.05	39.47	76.01	76.01	45.13	78.04
TPSA	90.9	77.76	164.75	38.77	141.34	41.93	97.99	111.13	111.13	57.53	131.36
Lipinski #violations	0	0	1	0	0	0	0	0	0	0	0
Ghose #violations	0	0	1	0	0	0	2	0	0	0	0
Veber #violations	0	0	1	0	0	0	0	0	0	0	0
Egan #violations	0	0	1	0	1	0	0	0	0	0	0
Muegge #violations	0	1	2	0	0	0	1	0	0	1	0
PAINS #alerts	0	1	1	0	1	0	1	0	1	0	1
Brenk #alerts	0	2	2	0	3	1	1	0	1	1	1
Leadlikeness #violations	0	1	1	2	0	0	1	0	0	1	0
Synthetic Accessibility	2.96	1.81	4.16	3.62	3.17	4.57	1.22	3.14	3.02	1.61	3.23

MW: Molecular weight; MR: Molar refractive; TPSA: Topological polar surface area.

## Data Availability

Data available on request.

## Supplementary Materials

The following are available online. Figure S1: Screening hypothesis is generated by a structure-based e-pharmacophore model consisting of one hydrogen bond acceptor (A), one hydrogen bond donor (D) and two aromatic rings (R), Figure S2: Screening hypothesis is generated by a structure-based e-pharmacophore model consisting of two hydrogen bond acceptor (A), one hydrogen bond donor (D) and three aromatic rings (R), Figure S3: ROC Plot between percent screen and percent actives found, Figure S4: ROC Plot between percent screen and true positive rate, Table S1: Report for Numeric Model kpls_radial_17 (AChE), Table S2: Report for Numeric Model pls_38 (BChE), Table S3: Report for Numeric Model kpls_desc_44 (MAO), Table S4: List of the different types of bonds with their respective interacting amino acids, that took part in the interaction between the three best ligands as well as controls and the target receptor, AChE, Table S5: List of the different types of bonds with their respective interacting amino acids, that took part in the interaction between the three best ligands as well as controls and the target receptor, BuChE, Table S6: List of the different types of bonds with their respective interacting amino acids, that took part in the interaction between the three best ligands as well as controls and the target receptor, MAO, Table S7: admeSAR prediction test compounds, Table S8: Toxicity Prediction test compounds.

## Sample Availability

Not available.
