# Supplementary material for: Deciphering the Interactions of Bioactive Compounds in Selected Traditional Medicinal Plants against Alzheimer’s Diseases via Pharmacophore Modeling, Auto-QSAR, and Molecular Docking Approaches
Source: Molecules. 2021 Apr 1;26(7):1996. doi: 10.3390/molecules26071996 (PMC8037217; doi:10.3390/molecules26071996)
Supplement: Supplementary file 1 [file molecules-26-01996-s001.pdf]

Deciphering the Interactions of Bioactive Compounds in Selected Traditional Medicinal plants against Alzheimer's diseases via Pharmacophore modeling, Auto-QSAR, and Molecular Docking Approaches

Oluwafemi Adeleke Ojo[1*], Adebola Busola Ojo[2], Charles Okolie[3], Mary-Ann Chinyere Nwakama [1], Matthew Iyobhebhe [1], Ikponmwosa Owen Evbuomwan[3], Charles Obiora Nwonuma[1], Rotdelmwa Filibus Maimako[1], Abayomi Emmanuel Adegboyega[4], Odunayo Anthonia Taiwo[5] Khalaf F. Alsharif[6] and Gaber El-Saber Batiha[7]

[1] Medicinal Biochemistry and Biochemical Toxicology Group, Department of Biochemistry, Landmark University, Omu-Aran, PMB 1001, Kwara State, Nigeria; charles.okolie@lmu.edu.ng (C.O); adamaryann62@gmail.com (M-A.C.N); iyobhebhematthew@gmail.com (M.I); nwonuma.charles@lmu.edu.ng (C.O.N); maimako.rotdelmwa@lmu.edu.ng (R.F.M).

[2] Department of Biochemistry, Faculty of Sciences, Ekiti State University, Ado-Ekiti, Nigeria; adebolaojo04@gmail.com.

[3] Department of Microbiology, Landmark University, Omu-Aran, PMB 1001, Kwara State, Nigeria

[4] Department of Biochemistry, Faculty of Basic Medical Science, University of Jos, Jos, Nigeria; abayomiadegboyega5@gmail.com

[5] Department of Biochemistry, Chrisland University, Abeokuta, Nigeria; odunayotaiwo25@gmail.com

[6] Department of Clinical Laboratory Sciences. College of Applied Medical Sciences, Taif University, P.O.Box 11099, Taif 21944, Saudi Arabia; alsharif@tu.edu.sa.

[7] Department of Pharmacology and Therapeutics, Faculty of Veterinary Medicine, Damanhour University, Damanhour 22511, AlBeheira, Egypt; gaberbatiha@gmail.com

* Correspondence: (OAO) ojo.adeleke@lmu.edu.ng; Phone no: +2347037824647

Table S1. Report for Numeric Model kpls_radial_17 (AChE)

ID	SetY	(Obs)	Y(Pred)	Error	Name
1	train	6.8300	6.6067	-0.2233	Compound 1
2	test	4.1700	4.4405	0.2705	Compound 3
3	train	4.1700	4.4405	0.2705	Compound 3
4	test	6.1000	6.0081	-0.0919	Compound 4
5	train	4.6800	4.7946	0.1146	Compound 5
6	train	4.6800	4.8012	0.1212	Compound 5
7	train	6.4100	6.3914	-0.0186	Compound 9
8	train	5.0100	5.0143	0.0043	Compound 10
9	test	5.0100	5.0143	0.0043	Compound 10
10	train	6.4100	7.0384	0.6284	Compound 13
11	test	4.1900	5.0712	0.8812	Compound 14
12	train	6.1200	5.9780	-0.1420	Compound 15
13	test	7.8500	7.5791	-0.2709	Compound 16
14	train	7.8500	7.6571	-0.1929	Compound 16
15	test	4.7300	5.1130	0.3830	Compound 17
16	train	4.4600	4.6881	0.2281	Compound 19
17	train	5.2900	5.2925	0.0025	Compound 20
18	test	6.2700	6.0297	-0.2403	Compound 21
19	train	6.8100	6.7701	-0.0399	Compound 24
20	test	6.7200	5.9345	-0.7855	Compound 25
21	train	6.1500	6.2667	0.1167	Compound 26
22	train	5.4200	4.8148	-0.6052	Compound 27
23	train	8.0300	8.4734	0.4434	Compound 32
24	train	9.6000	9.5475	-0.0525	Compound 36
25	train	6.7000	6.4662	-0.2338	Compound 41
26	train	5.4700	5.2669	-0.2031	Compound 39
27	train	4.4000	4.0505	-0.3495	Compound 42
28	train	4.3000	4.3258	0.0258	Compound 43
29	train	6.5500	7.3868	0.8368	Compound 44
30	train	5.2800	4.5731	-0.7069	Compound 45
31	train	6.9200	6.5827	-0.3373	Compound 46
32	test	6.4900	5.7909	-0.6991	Compound 47
33	test	4.6700	5.6772	1.0072	Compound 48
34	test	5.4800	5.6995	0.2195	Compound 49
35	train	7.7700	7.2043	-0.5657	Compound 50
36	train	6.5700	6.5637	-0.0063	Compound 52
37	train	6.5700	6.5637	-0.0063	Compound 52
38	train	7.4400	7.7771	0.3371	Compound 53
39	test	8.0500	7.6511	-0.3989	Compound 54
40	train	4.4700	4.4779	0.0079	Compound 55
41	train	5.9600	6.9058	0.9458	Compound 56
42	test	5.9600	7.0083	1.0483	Compound 56
43	train	7.8900	7.3378	-0.5522	Compound 57
44	train	5.5900	5.6069	0.0169	Compound 58
45	train	6.1000	5.8328	-0.2672	Compound 59
46	train	6.1000	5.8038	-0.2962	Compound 59
47	test	5.6000	5.9705	0.3705	Compound 60
48	train	5.6000	5.9677	0.3677	Compound 60
49	train	7.0300	6.9782	-0.0518	Compound 64
50	train	7.0300	6.9782	-0.0518	Compound 64

51	train	4.9300	6.0672	1.1372	Compound 65
52	train	5.5000	6.3753	0.8753	Compound 68
53	train	6.0500	6.1567	0.1067	Compound 70
54	train	6.6800	5.6077	-1.0723	Compound 71
55	train	5.3600	5.5218	0.1618	Compound 73
56	train	6.0500	5.9258	-0.1242	Compound 74
57	train	5.3600	5.1600	-0.2000	Compound 76
58	train	7.5100	7.3636	-0.1464	Compound 78
59	train	7.4100	7.0051	-0.4049	Compound 79
60	train	7.4100	7.0051	-0.4049	Compound 79
61	train	7.8000	7.0067	-0.7933	Compound 80
62	train	5.7300	5.8822	0.1521	Compound 82
63	test	5.7300	6.0230	0.2930	Compound 82
64	train	4.3600	5.3872	1.0272	Compound 83
65	test	6.9200	6.2054	-0.7146	Compound 84
66	test	7.1000	6.2631	-0.8369	Compound 85
67	train	6.0500	5.9840	-0.0660	Compound 86
68	train	4.5200	5.4614	0.9414	Compound 87
69	test	6.6200	6.4352	-0.1848	Compound 89
70	train	6.1000	6.3364	0.2364	Compound 90
71	train	6.0900	6.1060	0.0160	Compound 93
72	train	9.4800	8.8566	-0.6234	Compound 94
73	train	5.9200	5.4795	-0.4405	Compound 97
74	train	6.5200	6.5767	0.0567	Compound 99

Ranking score = 0.693689

Training Set		Test Set	
S.D.	R^2	RMSE	Q^2
0.4677	0.8602	0.5788	0.7307

Optimum number of factors = 2

Table S2. Report for Numeric Model pls_38 (BChE)

ID	Set	Y(Obs)	Y(Pred)	Error	Name
1	train	5.0700	5.2737	0.2037	Compound 2
2	train	7.3200	7.4471	0.1271	Compound 7
3	test	5.3500	5.9805	0.6305	Compound 9
4	test	7.5800	6.5828	-0.9972	Compound 11
5	train	7.5800	7.0026	-0.5774	Compound 11
6	train	6.4600	5.7392	-0.7208	Compound 12
7	train	5.3000	5.5860	0.2860	Compound 14
8	train	5.3000	6.0079	0.7079	Compound 14
9	test	5.1000	4.9656	-0.1344	Compound 17
10	train	5.1000	5.3410	0.2410	Compound 17
11	train	5.3300	5.5803	0.2503	Compound 22
12	test	6.7100	6.9142	0.2042	Compound 23
13	train	6.1000	6.4463	0.3463	Compound 24
14	train	6.2900	6.3338	0.0438	Compound 27
15	train	5.5100	5.9518	0.4418	Compound 30
16	test	5.5100	5.8733	0.3633	Compound 30
17	train	7.8200	7.2488	-0.5712	Compound 31
18	train	7.4000	7.4993	0.0993	Compound 32
19	train	5.8400	5.6192	-0.2208	Compound 33
20	test	8.0200	7.1981	-0.8219	Compound 35
21	train	7.9200	7.0999	-0.8201	Compound 37
22	train	5.7200	4.6638	-1.0562	Compound 49
23	test	5.5700	4.9310	-0.6390	Compound 54
24	train	4.6400	4.2273	-0.4127	Compound 55
25	test	5.0400	5.1152	0.0752	Compound 57
26	train	5.0400	4.8504	-0.1896	Compound 57
27	train	5.9500	6.9274	0.9774	Compound 59
28	train	5.5100	5.4517	-0.0583	Compound 61
29	train	4.7600	5.2424	0.4824	Compound 63
30	test	6.2900	5.8604	-0.4296	Compound 64
31	train	6.2900	6.3798	0.0898	Compound 64
32	train	6.0700	5.8583	-0.2117	Compound 72
33	train	5.7000	6.1409	0.4409	Compound 73
34	train	5.7500	5.5528	-0.1972	Compound 75
35	train	7.0300	6.9396	-0.0904	Compound 82
36	test	7.5200	6.9265	-0.5935	Compound 83
37	train	7.5200	6.7775	-0.7425	Compound 83
38	train	5.4100	5.5335	0.1235	Compound 84
39	train	4.4600	4.8961	0.4361	Compound 90
40	train	6.8300	6.7584	-0.0716	Compound 91
41	test	6.0400	5.9689	-0.0711	Compound 93
42	train	6.0400	5.8844	-0.1556	Compound 93
43	train	6.6000	6.6204	0.0204	Compound 96
44	train	4.4400	5.0855	0.6455	Compound 103
45	train	4.8800	4.0263	-0.8537	Compound 107
46	train	5.0900	5.3864	0.2964	Compound 113
47	train	4.0500	4.7404	0.6904	Compound 114

Ranking score = 0.682337

Training Set		Test Set	
S.D.	R^2	RMSE	Q^2
0.5091	0.7746	0.5406	0.7174

Optimum number of factors = 3

Table S3. Report for Numeric Model kpls_desc_44 (MAO)

ID	Set	Y(Obs)	Y(Pred)	Error	Name
1	test	8.0500	8.4662	0.4162	Compound 1
2	test	7.8500	6.9668	-0.8832	Compound 2
3	train	7.8500	8.0120	0.1620	Compound 2
4	train	4.5200	4.2258	-0.2942	Compound 3
5	train	4.6200	4.4534	-0.1666	Compound 5
6	train	5.8200	5.9741	0.1541	Compound 6
7	train	5.9700	5.9611	-0.0089	Compound 8
8	test	4.3000	4.4859	0.1859	Compound 9
9	train	6.2800	6.3942	0.1142	Compound 10
10	train	8.2000	7.8075	-0.3925	Compound 11
11	train	8.8300	8.6207	-0.2093	Compound 12
12	test	8.4100	8.0927	-0.3173	Compound 13
13	train	5.3200	6.0600	0.7400	Compound 15
14	test	7.2500	7.1778	-0.0722	Compound 16
15	train	8.0800	7.4883	-0.5917	Compound 17
16	test	4.1300	5.1916	1.0616	Compound 18
17	train	5.1200	4.3418	-0.7782	Compound 21
18	train	6.1900	5.7054	-0.4846	Compound 20
19	train	5.4400	5.8513	0.4113	Compound 25
20	train	5.6500	5.3474	-0.3026	Compound 27
21	train	7.1900	7.4145	0.2245	Compound 30
22	train	7.7000	7.3590	-0.3410	Compound 31
23	test	5.9200	5.8089	-0.1111	Compound 33
24	train	6.3100	7.2794	0.9694	Compound 34
25	train	8.1000	8.1536	0.0536	Compound 35
26	test	8.8300	8.5633	-0.2667	Compound 36
27	test	5.0500	6.0140	0.9640	Compound 37
28	train	7.3500	7.4074	0.0574	Compound 38
29	train	6.0300	6.7697	0.7397	Compound 39
30	train	4.1900	4.6223	0.4322	Compound 41
31	train	4.1900	4.3326	0.1426	Compound 41
32	train	5.5600	5.4238	-0.1362	Compound 43
33	train	6.7000	6.0072	-0.6928	Compound 48
34	train	8.7500	8.3541	-0.3959	Compound 49
35	train	7.0100	6.8285	-0.1815	Compound 50
36	test	7.7200	7.3274	-0.3926	Compound 53
37	test	6.6000	6.3355	-0.2645	Compound 57
38	train	4.5200	5.1886	0.6686	Compound 56
39	train	8.0600	8.1439	0.0839	Compound 58
40	test	5.4800	5.7527	0.2727	Compound 60
41	train	4.0400	3.6727	-0.3673	Compound 63
42	train	5.0600	5.5119	0.4519	Compound 64
43	train	6.0900	6.8697	0.7797	Compound 66
44	train	6.0900	6.3778	0.2878	Compound 66
45	train	7.7000	6.9517	-0.7483	Compound 67
46	train	7.7000	7.3785	-0.3215	Compound 67
47	train	7.8500	7.5407	-0.3093	Compound 68
48	train	7.8500	7.0781	-0.7719	Compound 68
49	train	5.1700	4.8958	-0.2742	Compound 69

50	test	5.1700	5.0669	-0.1031	Compound 69
51	train	6.3500	6.2436	-0.1064	Compound 70
52	train	4.7300	5.7490	1.0190	Compound 71
53	train	5.5300	6.1923	0.6623	Compound 74
54	train	4.5500	6.1653	1.6153	Compound 77
55	test	6.2300	6.0161	-0.2139	Compound 81
56	test	4.7000	5.9562	1.2562	Compound 82
57	train	8.8000	7.6816	-1.1184	Compound 88
58	train	7.7600	8.0244	0.2644	Compound 89
59	test	6.8400	5.9818	-0.8582	Compound 91
60	train	7.4500	7.2265	-0.2235	Compound 96
61	train	7.4500	6.7487	-0.7013	Compound 96
62	train	8.4200	8.4886	0.0686	Compound 97
63	train	8.2900	7.3503	-0.9397	Compound 99
64	train	5.8500	5.3310	-0.5190	Compound 100
65	train	4.2100	5.7577	1.5477	Compound 101
66	train	4.8100	4.4444	-0.3656	Compound 105
67	train	4.5000	3.5595	-0.9405	Compound 110
68	train	4.9800	6.0754	1.0954	Compound 111
69	train	6.0700	6.6665	0.5965	Compound 113
70	train	6.2400	5.5807	-0.6593	Compound 116
71	test	5.6200	6.4146	0.7946	Compound 118

Ranking score = 0.808180

Training Set		Test Set	
S.D.	R^2	RMSE	Q^2
0.6447	0.8132	0.6200	0.8130

Optimum number of factors = 4

Table S4. List of the different types of bonds with their respective interacting amino acids, that took part in the interaction between the three best ligands as well as controls and the target receptor, AChE.

Name of Ligand	Interacting amino acids	H-bond	C-H bond	Pi-pi stacking	π-alkyl
**Donepezil**	GLU A:202, GLY A:448, GLY A:121, HIS A:447, PHE A:338, PHE A:297, PHE A:295, VAL A:294, ARG A:296, SER A:293, LEU A:289, TYR A:341, TRP A:286, TRP A:86, TYR A:337 and TYR A:124	PHE A:295, ARG A:296	TYR A:341	TRP A:86, TRP A:286 and TYR A:337	TYR A:124
**Galanthamine**	GLY A:342, GLU A:292, TYR A:72, LEU A:76, PHE A:295, VAL A:294, ARG A:296, LEU A:289, SER A:293, PHE A:338, PHE A:297, and TRP A:286	-	SER A:293	TRP A:286	PHE A:338, PHE A:297
**Apigenin**	VAL A:294, PHE A:295, ASP A:74, TRP A:86, PHE A:338, PHE A:297, HIS A:447, SER A:203, GLY A:121, GLY A:122, GLY A:120 and ALA A:204	SER A:293, TYR A:337	-	TRP A:286, TYR A:341 and TYR A:124	-
**Luteolin**	HIS A:447, SER A:203, GLY A:122, PHE A:297, VAL A:294, LEU A:289, ASP A:74 and TRP A:86	GLY A:121, ARG A:296, PHE A:295, SER A:293 and TYR A:337	-	TYR A:124, PHE A:338, TYR A:341 and TRP A:286	-
**Ellagic acid**	TYR A:72, LEU A:76, ASP A:74, TYR A:124, TYR A:337, PHE A:297, PHE A:338, ARG A:296, VAL A:294, LEU A:289, SER A:293 and GLU A:292	PHE A:295	-	TRP A:286 and TYR A:341	-

Table S5. List of the different types of bonds with their respective interacting amino acids, that took part in the interaction between the three best ligands as well as controls and the target receptor, BuChE.

Name of Ligand	Interacting amino acids	H-bond	C-H bond	Pi-pi stacking	π-alkyl	π-sigma
**Donepezil**	GLY A:439, ILE A:442, GLY A:116, GLY A:117, SER A:198, PHE A:398, VAL A:288, THR A:120, TYR A:332 and ASP A:70	PHE A:295, ARG A:296	-	PHE A:329 and TRP A:231	ALA A:328, TRP A:430, TYR A:440, MET A:437, HIS A:438 and LEU A:286	TRP A:82
**Galanthamine**	THR A:120, GLY A:117, LEU A:286, GLY A:116, GLY A:439, GLU A:197, GLY A:115 and TYR A:128	SER A:198	-	PHE A:329	HIS A:438, PHE A:398	TRP A:82
**Quercetin**	TYR A:440, GLY A:439, SER A:198, ILE A:442, GLY A:115, TYR A:128, GLY A:116, ILE A:69, PRO A:84 and TYR A:332	ASP A:70, ASN A:68, ASN A:83, HIS A:438 and GLU A:197	-	TRP A:82	-	THR A:120
**Luteolin**	PRO A:84, GLN A:67, ILE A:69, ASP A:70, GLY A:116, TYR A:114, TYR A:128, SER A:198, GLY A:115, GLU A:197, GLY A:439, ILE A:442 and TYR A:440	ASN A:68 and HIS A:438	ASN A:83	TRP A:82	-	THR A:120
**Ellagic acid**	ASP A:70, TYR A:332, TRP A:430, ALA A:328, GLY A:439, SER A:198, ILE A:442, GLY A:115, TYR A:128, TYR A:114, GLY A:116 and GLY A:121	GLU A:197	HIS A:438	TRP A:82	-	-

Table S6. List of the different types of bonds with their respective interacting amino acids, that took part in the interaction between the three best ligands as well as controls and the target receptor, MAO.

Name of Ligand	Interacting amino acids	H-bond	C-H bond	Pi-pi stacking	π-alkyl	π-sulfur
**Donepezil**	THR A:426, GLY A:58, SER A:59, LYS A:296, TYR A:60, TYR A:435, MET A:436, THR A:43, GLY A:40, GLY A:13 and SER A:15	-	TYR A:398, GLY A:434, CYS A:172, GLN A:206 and ILE A:198	ARG A:42, TYR A:326 and LEU A:171	ALA A:439, ILE A:14 and PHE A:343	-
**Galanthamine**	PRO A:102, PHE A:103, THR A:196, THR A:478, THR A:195, ARG A:120, TRP A:119, THR A:479 and HIS A:115	ASN A:116	GLU A:483	-	TYR A:122	-
**Chlorogenic**	ILE A:14, THR A:43, MET A:436, GLY A:58, TYR A:435, GLY A:205, TYR A:60, SER A:59, GLY A:434, ALA A:439, GLY A:425, ALA A:263, ILE A:264, PRO A:265, GLY A:13 and GLY A:41	GLN A:206, THR A:426 and GLY A:40	SER A:15 and ARG A:42	TYR A:398	-	-
**Apigenin**	GLY A:13, THR A:43, GLY A:434, VAL A:61, GLN A:65, TYR A:60, SER A:59, PHE A:343, TYR A:398, GLY A:58 and THR A:426	ASN A:68 and HIS A:438	-	-	ALA A:439, ILE A:14, ARG A:42, MET A:436	CYS A:397
**Luteolin**	THR A:43, GLY A:58, SER A:59, TYR A:60, GLY A:434, THR A:426, GLY A:425 and GLY A:13	GLU A:197	-	-	ILE A:14, ALA A:439 and MET A:436	CYS A:397

Table S7. admeSAR prediction test compounds.

	Apigenin	Caffeic Acid	Chlorogenic acid	(R)-Donepezil	Ellagic acid	Galanthamine	Gallic acid	Kaempferol	Luteolin	P-coumaric	Quercetin
**Blood Brain Barrier**	-	-	-	+	-	+	-	-	-	-	-
**BRCP inhibitor**	+	-	-	-	-	-	-	+	+	-	+
**Biodegradation**	-	-	-	-	-	-	+	-	-	+	-
**BSEP inhibitor**	-	-	-	+	-	-	-	-	-	-	-
**Caco-2**	+	+	-	+	-	+	-	-	+	+	-
**CYP1A2 inhibition**	+	-	-	+	-	-	-	+	+	-	+
**CYP2C19 inhibition**	+	-	-	-	-	-	-	+	-	-	-
**CYP2C9 inhibition**	+	-	-	-	-	-	-	+	-	-	-
**CYP2C9 substrate**	-	-	+	-	-	-	-	-	-	-	-
**CYP2D6 inhibition**	-	-	-	+	-	-	-	-	-	-	-
**CYP2D6 substrate**	-	-	-	+	-	+	-	-	-	-	-
**CYP3A4 inhibition**	+	-	-	-	-	-	-	+	+	-	+
**CYP3A4 substrate**	-	-	+	+	-	+	-	+	-	-	+
**CYP inhibitory promiscuity**	+	-	-	+	-	-	-	+	+	-	+
**Human Intestinal Absorption**	+	+	+	+	+	+	+	+	+	+	+
**Human oral bioavailability**	-	+	-	+	+	+	+	-	-	-	-
**MATE1 inhibitor**	+	-	-	-	-	-	-	+	+	-	+
**Micronuclear**	+	+	+	-	+	-	+	+	+	+	+
**OATP1B1 inhibitor**	+	+	+	+	+	+	+	+	+	+	+
**OATP1B3 inhibitor**	+	+	+	+	+	+	+	-	+	+	+
**OATP2B1 inhibitor**	-	-	-	-	-	-	-	+	+	-	+
**OCT1 inhibitor**	-	-	-	+	-	-	-	-	-	-	-
**OCT2 inhibitor**	-	-	-	+	-	-	-	-	-	-	-

**P-glycoprotein inhibitor**	-	-	-	+	-	-	-	-	-	-	-
**P-glycoprotein substrate**	-	-	-	+	-	+	-	-	-	-	-
**Plasma protein binding**	0.95	0.78	0.75	0.97	0.99	0.50	0.67	1.06	1.04	0.72	1.17
**UGT catalyzed**	+	+	-	-	+	+	+	-	+	+	+

Table S8. Toxicity Prediction test compounds.

	**Apigenin**	**Caffeic Acid**	**Chlorogenic acid**	**(R)-Donepezil**	**Ellagic acid**	**Galanthamine**	**Gallic acid**	**Kaempferol**	**Luteolin**	**P-coumaric**	**Quercetin**
**Ames mutagenesis**	-	-	-	-	-	-	-	+	-	-	+
**Acute Oral Toxicity (c)**	III	IV	III	III	II	III	III	II	II	III	II
**Androgen receptor binding**	+	+	+	+	+	-	+	+	+	+	+
**Aromatase binding**	+	-	+	+	+	-	-	+	+	-	+
**Avian toxicity**	-	-	-	-	-	-	-	-	-	-	-
**Carcinogenicity (binary)**	-	-	-	-	-	-	-	-	-	-	-
**Carcinogenicity (trinary)**	Non-required	Non-required	Non-required	Non-required	Non-required	Non-required	Non-required	Non-required	Non-required	Non-required	Non-required
**Crustacea aquatic toxicity**	-	-	-	+	-	+	-	-	-	-	-
**Eye corrosion**	-	-	-	-	-	-	-	-	-	+	-
**Eye irritation**	+	+	-	-	+	-	+	+	+	+	+
**Estrogen receptor binding**	+	-	+	+	-	-	-	+	+	-	+
**Fish aquatic toxicity**	+	+	+	+	+	+	+	+	+	+	+
**Glucocorticoid receptor binding**	+	-	+	+	+	-	-	+	+	-	+
**Honey bee toxicity**	+	+	+	-	-	+	+	+	+	+	+
**Hepatotoxicity**	+	-	-	-	+	-	-	+	+	-	+

**Human either-a-go-go inhibition**	-	-	-	+	-	-	-	-	-	-	-
**Acute Oral Toxicity**	1.15	1.57	1.76	2.38	1.04	2.80	1.55	1.74	2.52	1.99	2.56
**Tetrahymena pyriformis**	1.23	0.10	0.68	1.66	1.66	0.94	0.69	0.82	1.14	-0.35	0.89
**Thyroid receptor binding**	+	-	-	+	-	+	-	+	+	-	+
**PPAR gamma**	+	+	+	-	+	-	-	+	+	-	+

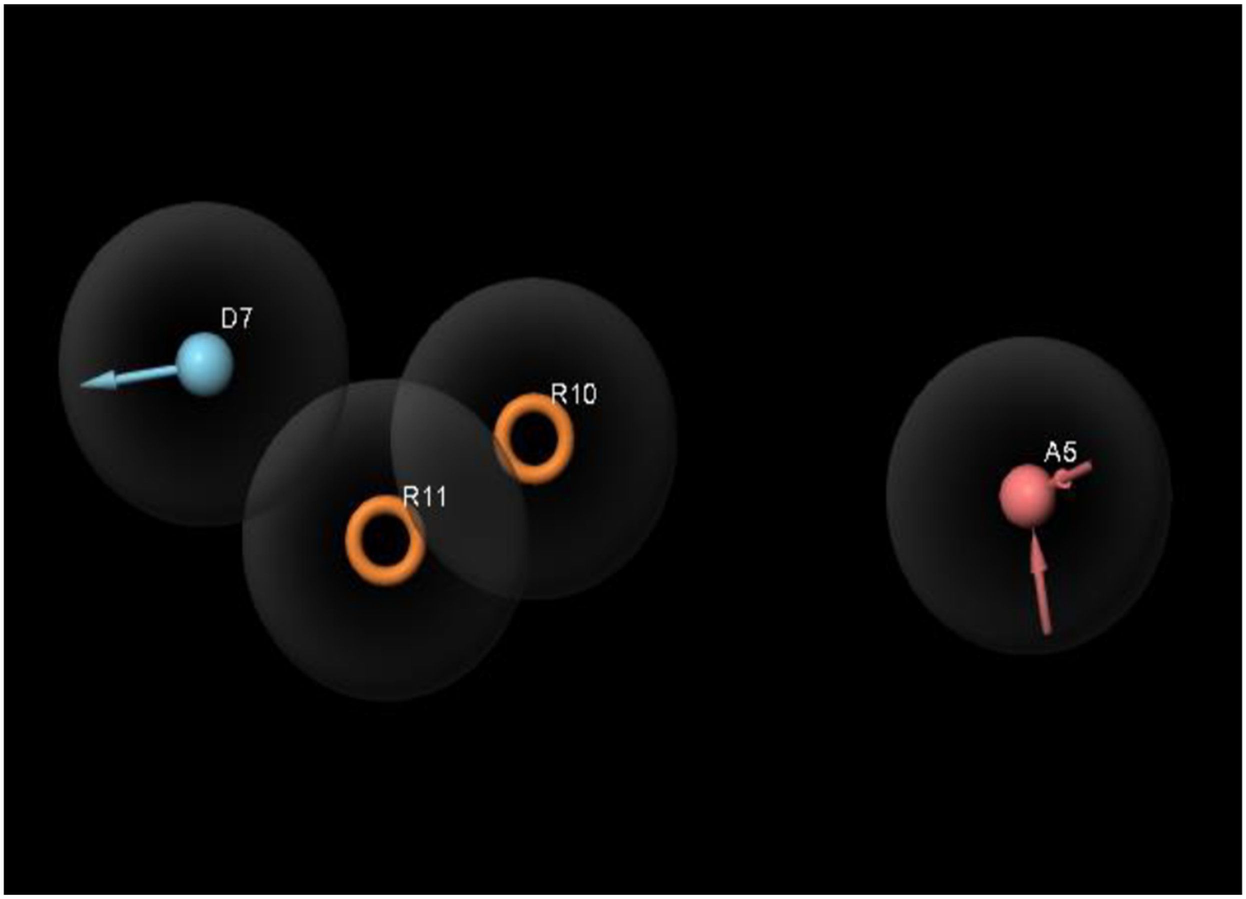

Figure S1. Screening hypothesis is generated by a structure-based e-pharmacophore model consisting of one hydrogen bond acceptor (A), one hydrogen bond donor (D) and two aromatic rings (R).

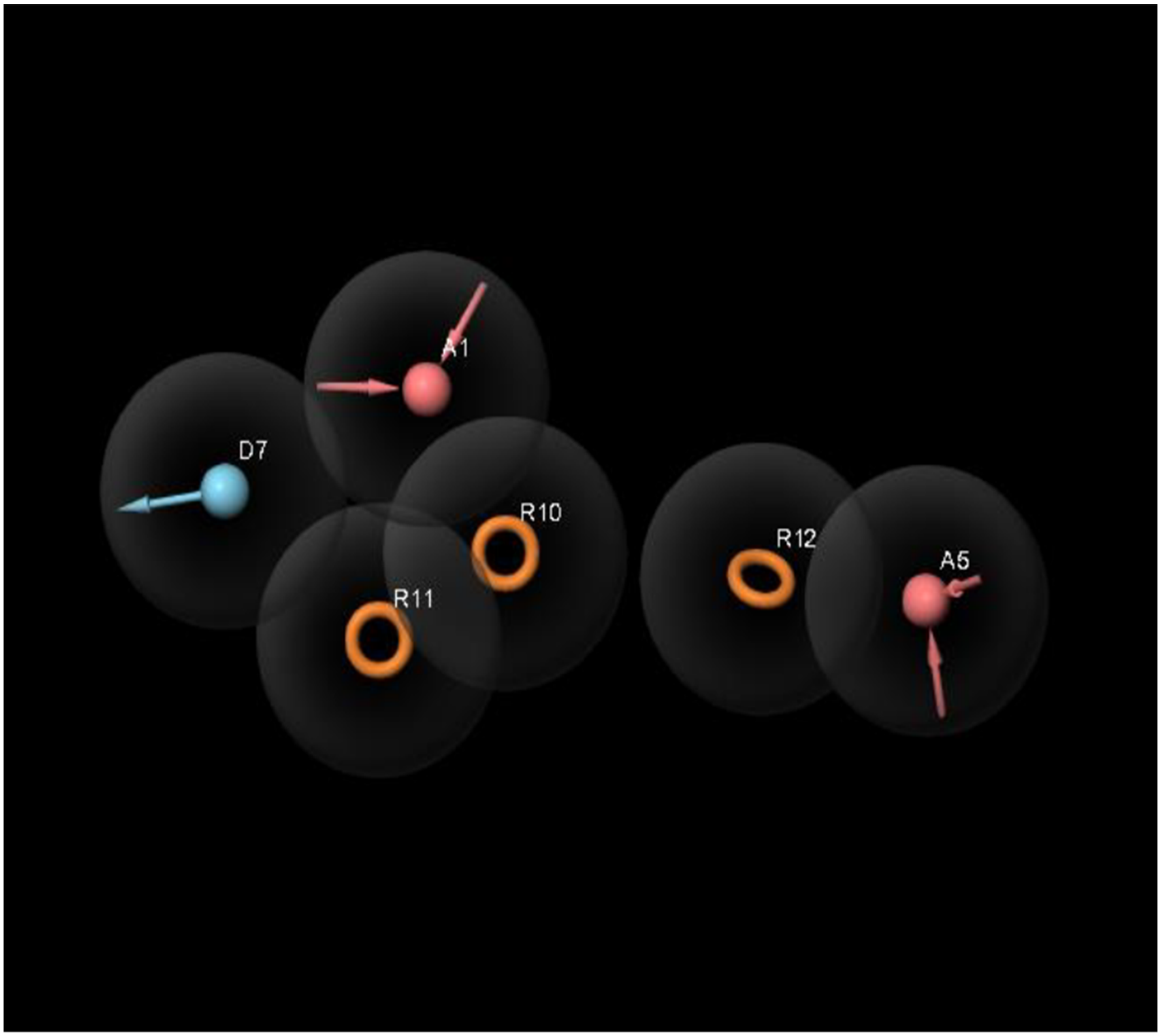

Figure S2. Screening hypothesis is generated by a structure-based e-pharmacophore model consisting of two hydrogen bond acceptor (A), one hydrogen bond donor (D) and three aromatic rings (R).

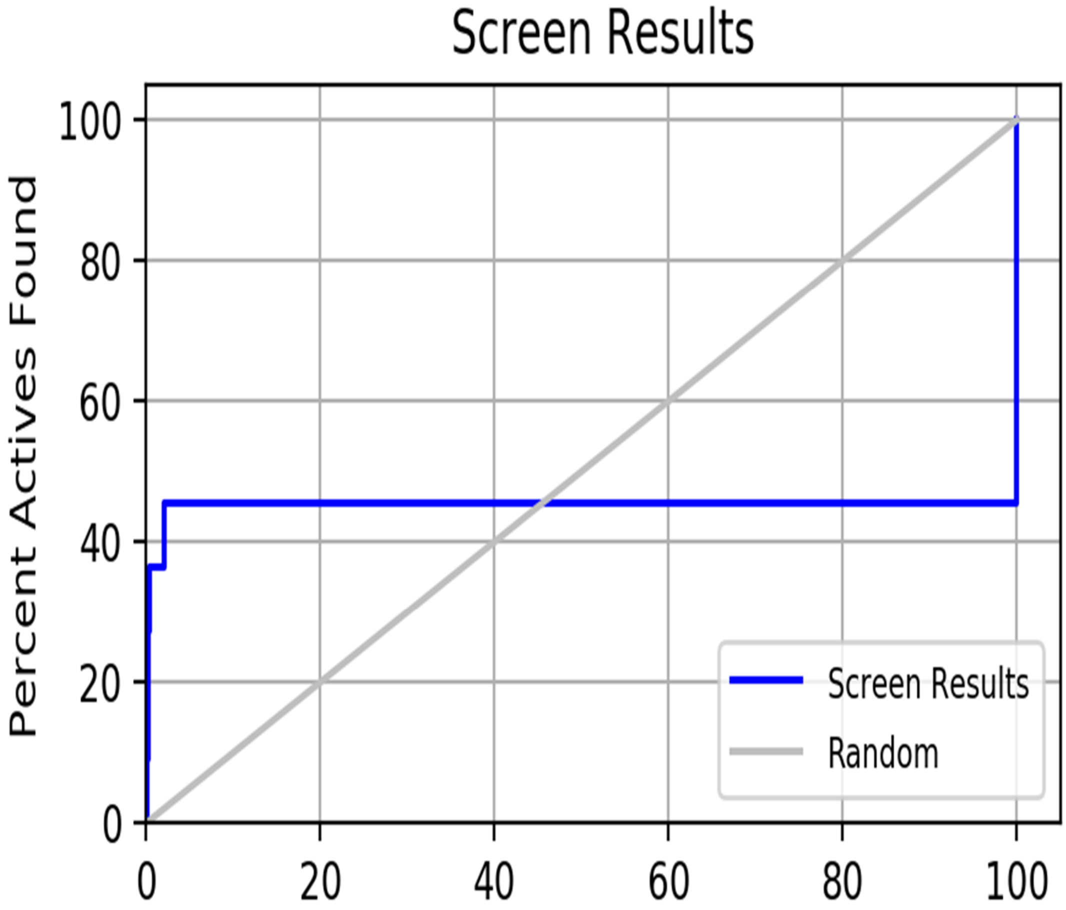

Figure S3: ROC Plot between percent screen and percent actives found

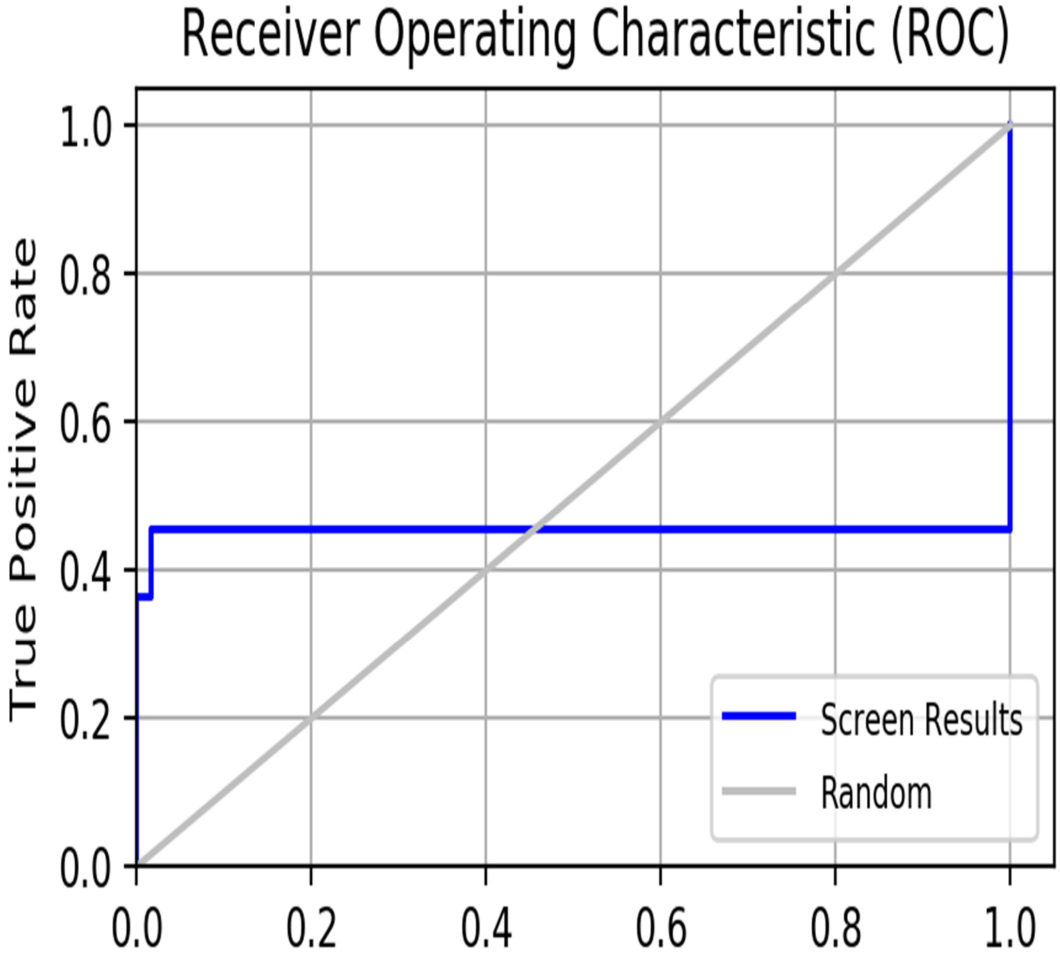

Figure S4: ROC Plot between percent screen and true positive rate
